# Protein-Based Model for Energy Transfer between Photosynthetic Light-Harvesting Complexes Is Constructed Using a Direct Protein–Protein Conjugation Strategy

**DOI:** 10.1021/jacs.3c02577

**Published:** 2023-07-13

**Authors:** Amanda J. Bischoff, Leo M. Hamerlynck, Amanda J. Li, Trevor D. Roberts, Naomi S. Ginsberg, Matthew B. Francis

**Affiliations:** † Department of Chemistry, University of California, Berkeley, California 94720, United States; ‡ Molecular Biophysics and Integrated Bioimaging Division, Lawrence Berkeley National Laboratory, Berkeley, California 94720, United States; § Department of Physics, University of California, Berkeley, California 94720, United States; ∥ Kavli Energy NanoScience Institute, Berkeley, California 94720, United States; ⊥ Materials Sciences Division, Lawrence Berkeley National Laboratory, Berkeley, California 94720, United States

## Abstract

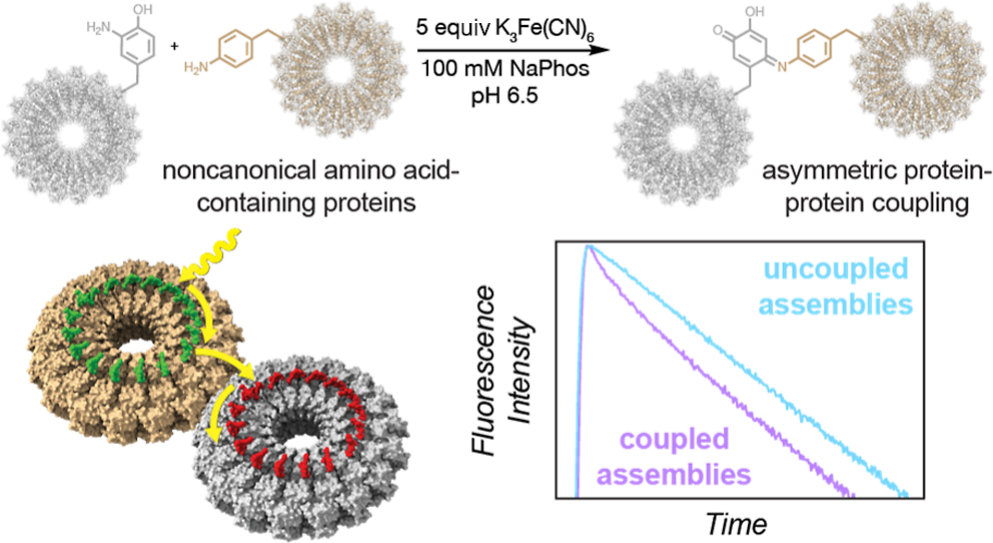

Photosynthetic organisms utilize dynamic and complex networks of pigments bound within light-harvesting complexes to transfer solar energy from antenna complexes to reaction centers. Understanding the principles underlying the efficiency of these energy transfer processes, and how they may be incorporated into artificial light-harvesting systems, is facilitated by the construction of easily tunable model systems. We describe a protein-based model to mimic directional energy transfer between light-harvesting complexes using a circular permutant of the tobacco mosaic virus coat protein (cpTMV), which self-assembles into a 34-monomer hollow disk. Two populations of cpTMV assemblies, one labeled with donor chromophores and another labeled with acceptor chromophores, were coupled using a direct protein–protein bioconjugation method. Using potassium ferricyanide as an oxidant, assemblies containing *o*-aminotyrosine were activated toward the addition of assemblies containing *p*-aminophenylalanine. Both of these noncanonical amino acids were introduced into the cpTMV monomers through amber codon suppression. This coupling strategy has the advantages of directly, irreversibly, and site-selectively coupling donor with acceptor protein assemblies and avoids cross-reactivity with native amino acids and undesired donor–donor or acceptor–acceptor combinations. The coupled donor–acceptor model was shown to transfer energy from an antenna disk containing donor chromophores to a downstream disk containing acceptor chromophores. This model ultimately provides a controllable and modifiable platform for understanding photosynthetic interassembly energy transfer and may lead to the design of more efficient functional light-harvesting materials.

## Introduction

**Figure 1:**
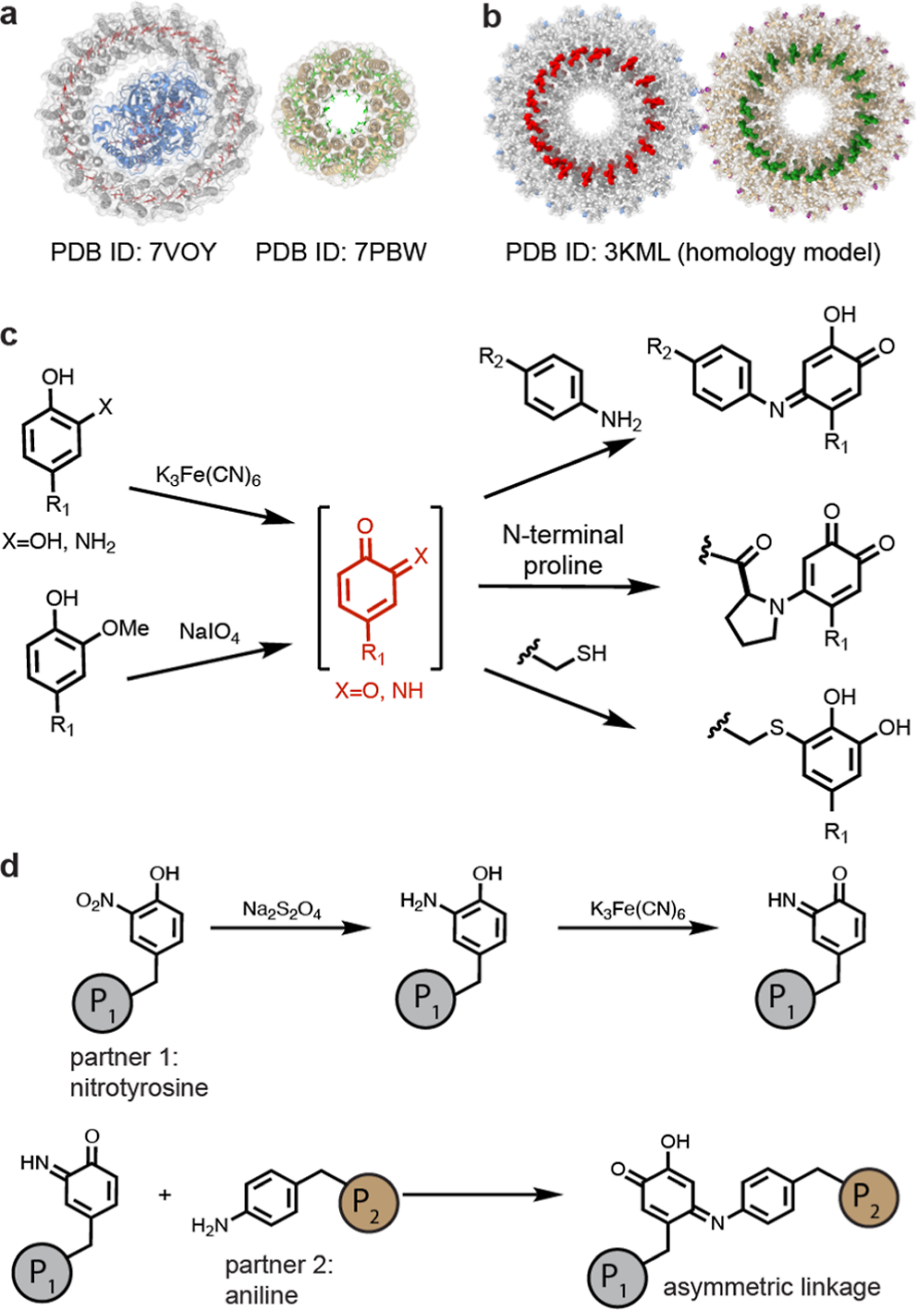
Strategy for site-selective protein–protein coupling. (a) A schematic of LH1 is shown next to LH2 from *R. sphaeroides*. Donor chromophores are shown in green, and acceptor chromophores are shown in red. (b) A mimic for LH2-to-LH1 energy transfer is envisaged using covalently conjugated cpTMV disks labeled with fluorescent dyes. Donor pigments are shown in green, and acceptor pigments are shown in red. (c) Oxidative coupling strategies can effect the chemoselective bioconjugation of *o*-quinoid intermediates to anilines, N-terminal proline residues, and thiols. (d) A scheme of the oxidative coupling strategy is shown for the asymmetric conjugation of two distinct, engineered protein assemblies containing noncanonical amino acids.

Within the membranes of photosynthetic organisms lie dynamic networks of multiple types of light-harvesting complexes, which are responsive to varying levels of light and environmental conditions.^1−3^ Many of these light-harvesting complexes are made up of identical repeating protein subunits embedded with photosynthetic pigments, which are often further embedded within lipid bilayers to form membrane-spanning complexes. Within photosynthetic membranes, light-harvesting complexes dynamically organize to form heterogeneous macroassemblies composed of antenna complexes that absorb light and transfer excited-state energy to complexes containing reaction centers. The rate and efficiency of processes in the energy transfer pathway and where energetic bottlenecks may occur differ among photosynthetic organisms. One such example is the pathway from light-harvesting complex 2 (LH2), which absorbs light energy and funnels it to light-harvesting complex 1 (LH1) and eventually to a reaction center (RC) in purple photosynthetic bacteria such as *R. sphaeroides* (Figure 1a). The inter-pigment distances between the nearest neighbors are low (∼1.0 nm in LH1 and ∼0.9 nm in LH2) with well-aligned transition dipole moments, leading to rapid and efficient energy transfer within single complexes.^4−6^ Energy transfer between light-harvesting complexes, such as from LH2 to LH2 or from LH2 to LH1, is not as well constrained and can occur across a range of distances as LH2 and LH1 are able to move laterally within the photosynthetic membrane. The lateral diffusion of LH2 and LH1 in the bilayer not only affects the interpigment distances between complexes and, therefore, energy transfer efficiency but also allows for the reorganization of complexes to respond to differing environmental conditions such as varying light intensity.^3^ Numerous studies have shown that the slow step in this process of energy transfer in purple photosynthetic bacteria is the LH1-to-RC transfer, likely due to the relatively large distances involved between bacteriochlorophyll pigments (3–4 nm).^7−10^ In contrast, 2D electronic spectroscopy has revealed that the transfer of energy between chlorosome antennae and the FMO complex in green sulfur bacteria occurs on a slower timescale than that between the FMO complex and RCs and on a much slower timescale than energy transfer within a subunit.^2,11^

These studies have made substantial progress toward understanding the timescales involved in photosynthesis. However, deconvolving the numerous processes influencing energy transfer efficiency *in vivo* or in isolated photosynthetic membranes presents a significant challenge due to additional components within the photosynthetic membrane and the environment and difficulty in isolating functional networks of associated light-harvesting complexes from their membranes. Previous studies using synthetic model systems have shed light on exciton transport across pigment compositions and distances analogous to those found in photosynthesis. This has been achieved using layered carbon nanotubes,^12^ supramolecular polymers,^13^ metallacycles,^14^ multicomponent self-assembling materials,^15^ and biomolecular frameworks.^16^ These self-assembling systems have demonstrated how light-harvesting capabilities may have arisen in primitive organisms from simple peptide, porphyrin, and mineral components.^17^ Providing a scaffold to confine chromophores within ordered structures in solution can also increase their photocatalytic ability while avoiding photodegradation.^18,19^ However, few of these scaffolds imitate the precise, discrete, and circular arrangement of chromophores present in many photosynthetic complexes such as LH1 and LH2. Understanding how these protein-bound chromophore assemblies interact to transfer energy across distances within photosynthetic membranes is an added challenge that is difficult to address in model systems.^12,20^

Herein, we develop a model system for the study of energy transfer between distinct light-harvesting complexes (LHCs) of differing pigment compositions using a circular permutant of the tobacco mosaic virus coat protein (cpTMV). cpTMV assembles into double-layered disks with a hollow pore, with 17 monomers per disk and 34 monomers per assembly, and its assembly state is stable across a broad pH and ionic strength range.^21^ Mutants and conjugates of cpTMV have been shown to maintain their assembly state after storage for several days at room temperature.^22^ The structure of cpTMV resembles the structure of light-harvesting complexes such as LH1 and LH2, which also assemble into flattened disks composed of identical monomers in circular arrays. cpTMV has previously been used as a tunable scaffold for the attachment of circular chromophore arrays, with varying constraints and interchromophore distances, to mimic single LHCs, providing insights into the physical characteristics underpinning photosynthetic energy transfer.^21,23−29^ The interpigment distances in pigment-labeled single cpTMV assemblies can be varied based on the attachment site on the protein to between 1.6 and 3.2 nm on average, slightly larger than those found in LH2 and LH1.^29^ Flexibility in the linkers between chromophores and the protein surface also introduce disorder in the orientation between chromophores attached to cpTMV, although this flexibility can be mitigated by attaching chromophores to the region between the two disks in single cpTMV assemblies.^28,29^

In this work, we use a bioconjugation strategy to appose complete assemblies of cpTMV disks that contain donor and acceptor chromophores in a controlled fashion, thus imitating the spatial relationships that foster intercomplex energy transfer between nonidentical light-harvesting complexes such as LH1 and LH2 (Figure 1a,b). LH1 and LH2 are found in complex membrane environments, and LH2 to LH1 energy transfer is difficult to study in isolation. The coupled cpTMV complex model with a single acceptor complex linked peripherally to donor complexes allows intercomplex energy transfer to be examined in aqueous solution, isolated from other photosynthetic components. The cpTMV assemblies were coupled by modifying an oxidative coupling strategy previously developed in our lab to bind two large protein assemblies directly and covalently. The multicomponent cpTMV-based systems demonstrate energy transfer between donor and acceptor complexes. Going forward, this strategy provides an adjustable solution-based model for studying photosynthetic energy transfer.

## Results and Discussion

To construct the protein scaffold for interassembly energy transfer, a method was required for covalently coupling two distinct donor and acceptor protein assemblies, each of which was composed of 34 monomers assembled noncovalently into a double-layered hollow disk. A promising method would modify a strategy previously developed by the Francis lab in which a catechol, aminophenol, or methoxyphenol is oxidized to an *o*-quinone or *o*-iminoquinone using potassium ferricyanide or sodium periodate as a small-molecule oxidant before coupling to thiol- or amine-based nucleophiles (Figure 1c).^30−32^ Herein, we expand the strategy to form direct, site-selective, and oxidative protein–protein linkages utilizing a pair of noncanonical amino acids (ncAAs), *p*-aminophenylalanine (*p*AF) and 3-nitrotyrosine (3NY), installed during protein expression (Figure 1d).

The first step in the creation of the donor–acceptor complex assemblies was the installation of ncAAs in peripheral sites on cpTMV disks. To select the sites for amino acid mutagenesis, we took inspiration from the work of the Wang laboratory, which engineered recombinant TMV to assemble into thin nanosheets of conjugated assemblies through engineered cysteine or histidine residues on and near the N-termini of the disks.^33,34^ We selected analogous sites on the cpTMV construct, which is a permutant of the wild type, for mutation to *p*AF and 3NY. These constructs were prepared using amber codon suppression, as reported previously.^35−39^ Screening across a variety of expression conditions and mutation sites revealed that the S65 site was most amenable to the installation of these ncAAs, though there was also significant yield at the S63 site (Figure S1). The cpTMV-S65-*p*AF mutant had also been previously shown to couple to phenol-modified gold nanoparticles using an enzymatic oxidative coupling method,^38^ suggesting that this site may be optimal for the conjugation of two large protein assemblies. Incorporation of the ncAA was verified by mass spectrometry (MS). Because tyrosine and *p*AF differ by only 2 Da and are therefore difficult to distinguish using protein MS, further verification that the correct amino acid had been installed was performed by omitting the ncAA from the expression media and analyzing the cell lysate using gel electrophoresis. This showed that full-length cpTMV expression did not occur when the ncAA was omitted from the protein expression media (Figure S1). The size of the assemblies formed from these constructs matched the previously reported cpTMV double-disk structures,^21^ at approximately 600 kDa as verified by size exclusion chromatography (SEC; Figure S2a) and 18 × 5 nm with the double disk with pore morphology as confirmed by transmission electron microscopy (TEM) of the cpTMV-S65-3NY construct (Figure S2b).

**Figure 2:**
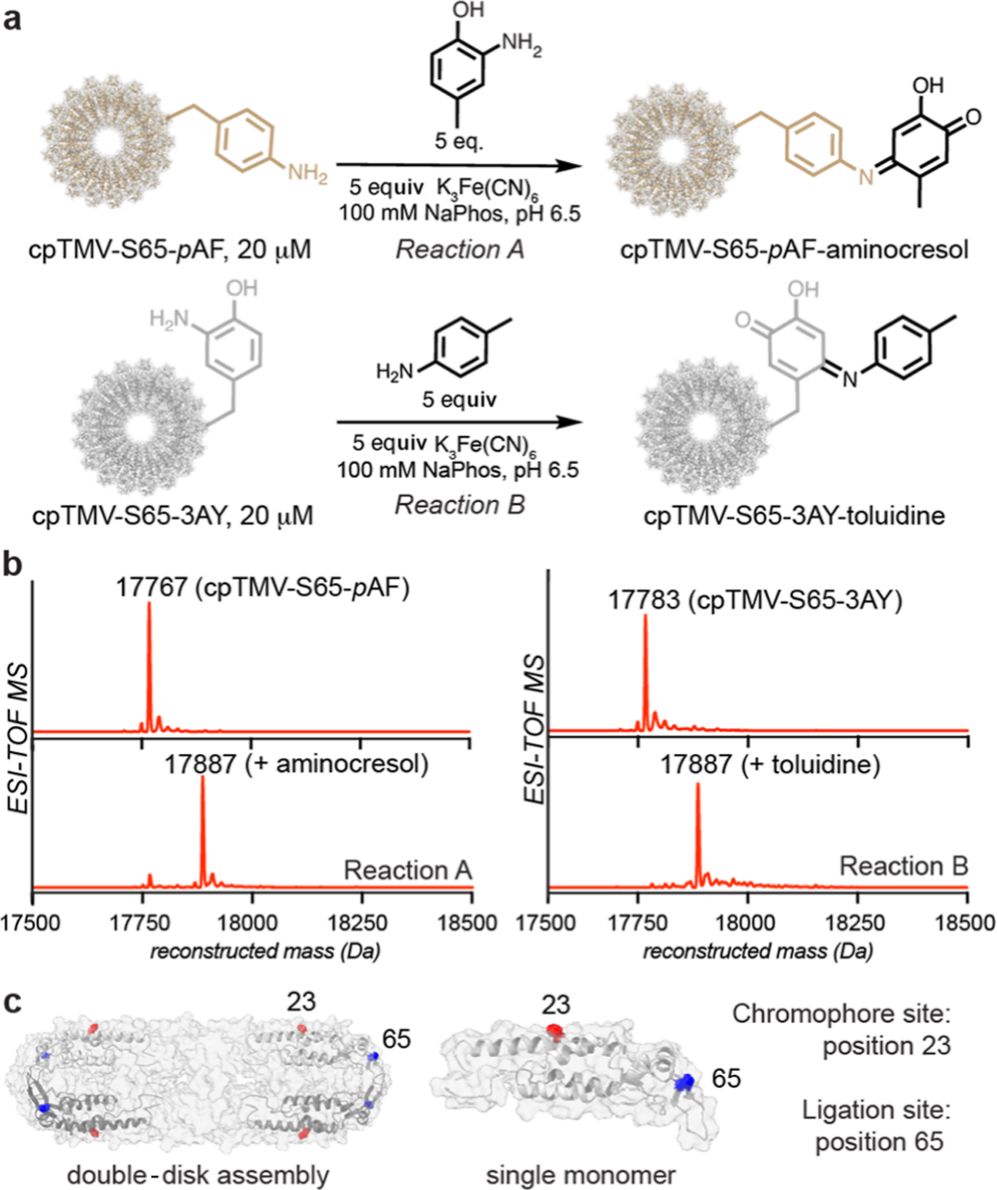
Chemical confirmations for accessibility and modification of ncAA-containing amino acids through small-molecule couplings. (a) Conditions are shown for K_3_Fe(CN)_6_-mediated oxidative coupling to both cpTMV-S65-*p*AF (reaction A) and cpTMV-S65-3AY (reaction B). (b) Reconstructed ESI-TOF mass spectra indicated high conversion of each ncAA-containing cpTMV monomer to the expected oxidative coupling product (expected MW: 17,887 Da). (c) A cutaway view is provided, showing cpTMV monomers on opposite sides of the disk in gray, sites for protein–protein conjugation in blue, and sites for pigment attachment in red. A close-up view of a single monomer of the individual double-disk assembly is also shown.

The cpTMV disks containing ncAAs were then subjected to potassium ferricyanide-mediated oxidative coupling conditions as previously reported by our laboratory for *p*AF- and 3NY-containing proteins (Figure 2a).^30−32^ The accessibility of the mutated amino acids in the cpTMV-S65-*p*AF and -3NY constructs was first examined through small-molecule couplings. For the 3NY mutant, treatment with sodium dithionite was first required to reduce the nitrophenol to an aminophenol, producing cpTMV-S65-3-aminophenol (cpTMV-S65-3AY). This reduction proceeded at 83% yield (Figure S3a,b), as measured by MS peak integration; however, some re-oxidation of the aminophenol may have occurred in air or during elution prior to the MS measurement. After removal of sodium dithionite, cpTMV-S65-3AY was directly added to a solution of *p*-toluidine and potassium ferricyanide, resulting in a single modification of each monomer to complete conversion (Figure 2b). This suggests that reduction of cpTMV-S65-3NY to cpTMV-S65-3AY proceeded to full conversion prior to the oxidative coupling reaction as no appreciable amount of unmodified protein was observed. Treating the *p*AF-containing variant with amino-*p*-cresol and potassium ferricyanide also resulted in single modification of each monomer to full conversion (Figure 2b).

For both the cpTMV-S65-*p*AF and cpTMV-S65-3AY constructs, full conversion of each monomer to form one oxidative coupling product (as shown in Figure 2b) results in 34 total modifications per double-disk assembly. Each of these constructs contains multiple reactive side chains including lysine, tyrosine, and cysteine, along with termini within the cpTMV pore. A construct identical in sequence to cpTMV-S65-*p*AF and cpTMV-S65-3AY but with serine in place of the ncAAs at position 65 (cpTMV-S65) showed no reactivity when subjected to the same oxidative conditions, indicating that only the engineered S65-ncAAs were modified, without cross-reactivity with other amino acids in the cpTMV sequence (Figure S4).

**Figure 3:**
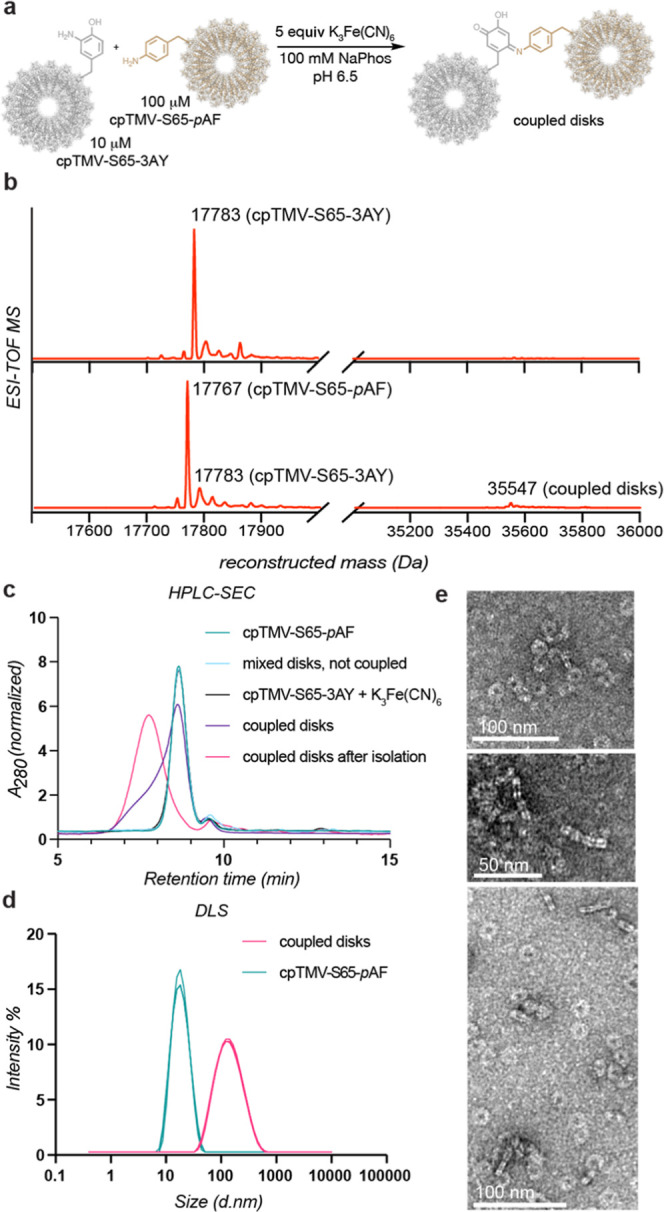
Asymmetric coupling of intact cpTMV assemblies. (a) A scheme shows the covalent oxidative coupling of noncanonical amino acids on separate cpTMV disk assemblies. (b) Mass spectrometry shows both reduction of cpTMV-S65-3NY to cpTMV-S65-3AY and coupling of the monomers of the limiting protein, cpTMV-S65-3AY, at an estimated 31% yield (expected MW: 35,547 Da). (c) A size increase after oxidative coupling was verified and assemblies were isolated using SEC. Omitting the oxidant or one of the coupling partners, cpTMV-S65-*p*AF, prevented the size increase. (d) The increase in size and isolation of assemblies from individual disks was verified by DLS. (e) TEM images show disks coupled on their periphery through face and side views of coupled assemblies. A collection of TEM images is found in Figure S6.

Following the confirmation of site selectivity, an oxidative conjugation of the two coupling partners, cpTMV-S65-*p*AF and cpTMV-S65-3AY, was performed (Figure 3a) by mixing the two in a ratio of 1:1 through 10:1 cpTMV-S65-*p*AF/cpTMV-S65-3AY. A higher stoichiometry of cpTMV-S65-*p*AF was used to promote the formation of smaller assemblies, rather than large sheets, of asymmetrically conjugated disks. When at a ratio of 10:1 of cpTMV-S65-*p*AF/cpTMV-S65-3AY, analysis of the mass spectrum resulted in an estimated modification of 31% of monomers of cpTMV-S65-3AY (the limiting coupling partner) or an average of 10.5 modifications per double-disk assembly. This does not necessarily demonstrate that the cpTMV-S65-3AY disks were coupled to 10 cpTMV-S65-*p*AF disks, as each disk contains 34 monomers, and it is likely that there were multiple oxidative coupling linkages between monomers in a single disk pair. Qualitative analysis using gel electrophoresis also showed formation of conjugates under the oxidative coupling conditions (Figures 3a,b and S5a). A series of controls showed that cpTMV-S65-*p*AF, cpTMV-S65-3AY, and K_3_Fe(CN)_6_ are all required to achieve appreciable disk–disk conjugation (Figure S5a). This indicates that the oxidative coupling reaction was asymmetric, with cpTMV-S65-*p*AF disks only reacting with cpTMV-S65-3AY disks and vice versa and no coupling of like disks observed.

The effect of the oxidative coupling conditions on the assembly state of cpTMV was next assessed. Decomposition of the self-assembled disks into monomers was not expected from the oxidative coupling conditions used because other virus-like particles have been shown to maintain their assembly state under similar oxidative conditions;^40,41^ however, we wanted to ensure that the potential strain placed on the noncovalent interactions between monomers by the close proximity to an adjacent large protein complex did not cause decomposition. The attachment of two intact cpTMV disks would result in a 1.2 MDa, 36 nm long complex, and even larger complexes may be expected from the conjugation of multiple disks. A size increase from individual to conjugated protein was observed through the appearance of a higher MW species using native gel electrophoresis (Figure S5b) and SEC, as revealed by a higher-molecular weight shoulder when compared to individual assemblies, and by dynamic light scattering (DLS), which also showed two distinct populations corresponding to individual disks and larger assemblies after coupling, suggesting an increase in size rather than decomposition to monomers upon conjugation. Isolation of the larger-sized complexes by SEC and size measurement by SEC and DLS showed a successful separation of the larger complexes from individual assemblies (Figure 3c,d). A comparison to several controls, including a control with cpTMV-S65-3AY and K_3_Fe(CN)_6_ but lacking cpTMV-S65-*p*AF, did not show evidence of a size increase and, therefore, conjugation of cpTMV-S65-3AY to itself, further confirming that this protein–protein coupling reaction occurs asymmetrically.

Disks were conjugated at an equal ratio of cpTMV-S65-*p*AF/cpTMV-S65-3AY at a concentration of 20 μM for visualization using TEM. Conjugated disks were observed at dilute concentrations (Figure 3e; full images and additional images are shown in Figure S6), whereas an uncoupled control did not display the same morphology (Figure S6). While many of the TEM images appeared to show the coupled cpTMV disks in a parallel orientation with respect to one another, suggesting that this conformation is preferred in the coupled assemblies, it is possible that there is flexibility in the protein–protein linkage, and some coupled assemblies may exhibit a less parallel orientation. With confirmation that the protein-based model scaffold had been successfully constructed, we moved on to attach synthetic dyes and examine the excited-state energy transfer properties of the multi-cpTMV assemblies.

**Figure 4:**
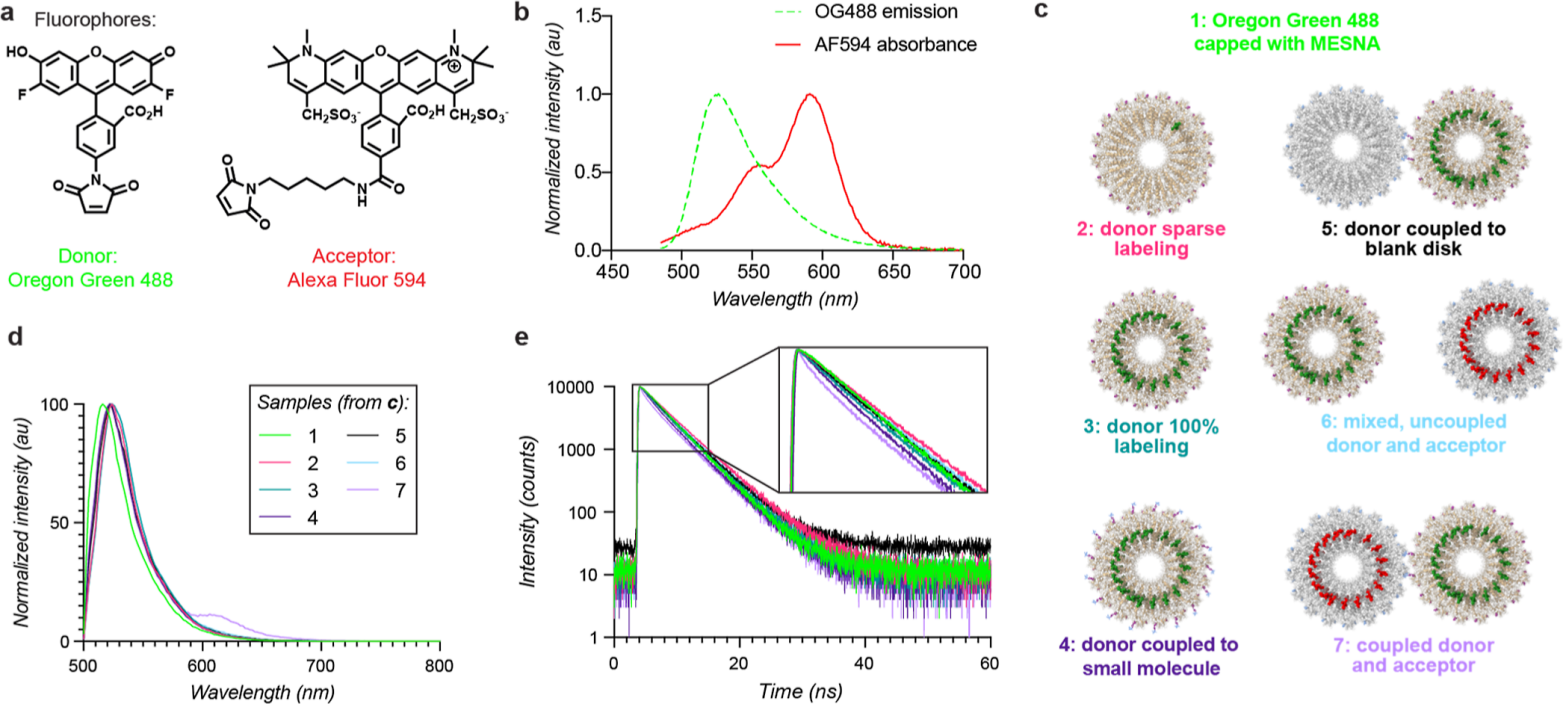
Energy transfer between conjugated disk assemblies. (a) The donor and acceptor pair of dyes, Oregon Green 488 and Alexa Fluor 594, are shown with maleimide handles for attachment to the protein surface. (b) The absorbance spectrum of Alexa Fluor 594 and emission spectrum of Oregon Green 488, both conjugated to cpTMV assemblies, show spectral overlap. (c) A diagram of oxidatively coupled assemblies and a subset of comparison populations are shown, including cpTMV-S65-*p*AF disks, both sparsely (**2**) and completely (**3**) labeled with OG488, a fully labeled cpTMV-S65-*p*AF disk oxidatively coupled to 4-methylcatechol (**4**), a fully labeled cpTMV-S65-*p*AF disk oxidatively coupled to a cpTMV-S65-3AY disk bearing no pigments (**5**), a mixture of fully donor-labeled cpTMV-S65-*p*AF disks and fully acceptor-labeled cpTMV-S65-3AY disks which were not coupled (**6**), and a fully donor-labeled cpTMV-S65-*p*AF disk oxidatively coupled to a fully acceptor-labeled cpTMV-S65-3AY disk (**7**). (d) Emission spectra of coupled assemblies at an excitation wavelength of 465 nm demonstrate that emission only occurs when the donor and acceptor disks are oxidatively coupled and not when they are simply mixed. The legend indicates samples depicted in (c). (e) The fluorescence lifetime of OG488 measured at 524 nm decays more rapidly for the coupled assemblies than for comparable controls; samples are the same as those shown in (c). The inlay shows a magnified version of early timepoints for better visualization of the shortened lifetime of sample **7** in mauve.

For the installation of light-harvesting chromophores, a cysteine was engineered on the exterior surface of each monomer in the cpTMV coat at the S23 position (Figure 2c), which has previously been used for chromophore attachment to cpTMV.^28^ The pigments selected for this study of energy transfer were Oregon green 488 maleimide (OG488) and Alexa Fluor 594 maleimide (AF594) due to their favorable spectral overlap for Förster resonance energy transfer (FRET), high extinction coefficients, and resistance to photobleaching (Figure 4a).^42^ These partners have previously been conjugated to TMV,^25^ can be selectively excited by tuning the wavelength of light used, and have high spectral overlap between the emission spectrum of the donor OG488 and the absorption spectrum of the acceptor AF594 (Figure 4b), enabling energy transfer between the two. The donor chromophores were conjugated to cpTMV-S65-*p*AF, and acceptor chromophores were conjugated to cpTMV-S65-3NY. Using these maleimide-modified dyes, near-quantitative labeling of all monomers per disk at position S23C was achieved, resulting in circular arrays of 17 dyes attached to the surface of the protein assembly. For a comparison without pigment–pigment interactions, cpTMV-S65-*p*AF was also labeled at a ratio of one dye per disk (Figure S7a,b).

Donor dye-labeled, *p*AF-containing cpTMV assemblies were then coupled to acceptor dye-labeled, 3AY-containing cpTMV assemblies at a ratio of 2:1 for fluorescence experiments. This coupling led to the formation of the desired donor–acceptor model **7** (Figures 4c and S8b). The constructs used to join the donor and acceptor disks were identical to those used for small-molecule labeling experiments (Figure 2a,b), with the exception of the S23C mutation for dye attachment. Because the dye attachment proceeded to full conversion and was performed before the disk coupling, all exposed cysteines in these constructs were expected to be protected by their conjugation to the maleimide-containing dyes (Figure S7b). Additionally, all other residues on these constructs were shown not to react under the oxidative coupling conditions in small-molecule conjugations (Figure S4). These factors indicate that the two disks in **7** were joined site-specifically at only positions S65-*p*AF on the donor disk and S65-3AY on the acceptor disk.

The energy transfer ability of donor pigment-containing cpTMV assemblies to acceptor pigment-containing assemblies (**7**) was then explored (Figure 4c). The pigment-labeled, conjugated complexes were compared to several controls in order to understand the contributions of surrounding solvents, proteins, and pigments on energy transfer. These controls included the OG488 maleimide dye capped with 2-mercaptoethanesulfonic acid (Mesna) (**1**), cpTMV disks with only sparse OG488 labeling (**2**), cpTMV disks with all monomers labeled with OG488 (**3**), cpTMV disks fully labeled with OG488 and coupled to 4-methylcatechol at the S65 site (**4**), cpTMV disks fully labeled with OG488 and coupled to a cpTMV disk with no chromophore labeling (**5**), and a mixture of cpTMV disks fully labeled with OG488 and cpTMV disks fully labeled with AF594, where the disks were not coupled (**6**) (Figures 4c and S7, S8). For samples **5** and **7**, coupled assemblies were separated from uncoupled disks using SEC, and the fractions containing coupled disks were isolated prior to fluorescence measurements. Spectroscopic measurements of each of samples **1**–**7** were taken at low concentrations (<2 μM dye) to avoid appreciable energy transfer between noncovalently tethered complexes. Emission spectra of each of these populations at an excitation wavelength of 465 nm, which is optimized to excite OG488 without exciting AF594, resulted in emission of the acceptor AF594 exclusively in the coupled donor and acceptor assemblies (**7**) (Figure 4d, mauve) but not when uncoupled donor and acceptor assemblies were mixed at the same concentration. A small amount of excitation of AF594 was observed in fully labeled acceptor samples, but direct excitation of the acceptor was not observed in controls containing both the donor and acceptor (Figure S9).

The fluorescence lifetime of the donor chromophore, OG488, was also measured for the coupled donor–acceptor assemblies and controls. The fluorescence lifetime of OG488 was shorter in the coupled assemblies than in each of the controls, particularly at shorter timescales (Figure 4e; individual spectra, the instrument response function, and fit residuals are shown in Figures S10–S16). A slight decrease in fluorescence lifetime also occurred for the donor disk when coupled to a small molecule (**4**) but not for the donor disk coupled to an unlabeled acceptor disk (**5**). While 91% of monomers of the donor disk were coupled to 4-methylcatechol in the small-molecule case, resulting in ∼31 oxidative coupling linkages per disk, the interdisk coupling resulted in only 3–4 linkages per disk (Figure S8). This shows that the oxidative coupling linker may provide an avenue for OG488 energy dissipation, but this does not appear significant for the small number of linkages present in coupled disks, as evidenced by the donor disk coupled to a blank disk case. Notably, the mixture of donor and acceptor disks in solution at the same concentration as coupled disks (**6**) did not demonstrate a decrease in the donor fluorescence lifetime when compared to donor disks alone, indicating that the decrease in the fluorescence lifetime in the coupled donor and acceptor assemblies (**7**) is primarily due to energy transfer between coupled donor and acceptor complexes rather than nearby complexes in solution.

To deepen our understanding of the interdisk energy transfer process observed, we deconvolved the fluorescence lifetimes into their constituent components using FluoFit software (Table 1). Without conjugation to cpTMV, OG488 maleimide capped with Mesna exhibited a decay well described by a monoexponential fit, with a fluorescence lifetime of 4.1 ns, in agreement with the canonical value of 4.1 ns. When conjugated to cpTMV, the fluorescence lifetime of OG488 was best described by two decay components in all cases. The long component of the fluorescence lifetime remained relatively consistent across samples, with the fluorescence lifetime being slightly increased by comparison to the free dye, indicating that this component describes energy dissipation *via* dye–solvent interactions.

**Table 1:** Fluorescence Lifetime Components of Coupled cpTMV Assemblies and Controls at 465 nm Excitation and 524 nm Emission

sample	long component (ns)	short component (ns)	short component % amplitude (%)
OG 488 alone (Mesna capped) (**1**)	4.11 ± 0.02^a^		
donor disk sparsely labeled with OG488 (**2**)	4.79 ± 0.02	2.0 ± 0.2	11.52
donor disk quantitatively labeled with OG488 (**3**)	4.52 ± 0.02	1.79 ± 0.09	21.50
donor disk quantitatively labeled with OG488, coupled to 4-methylcatechol (**4**)	4.36 ± 0.02	1.37 ± 0.07	28.69
donor disk quantitatively labeled with OG488, coupled to unlabeled acceptor disk (**5**)	4.52 ± 0.02	1.7 ± 0.1	15.69
donor disk quantitatively labeled with OG488, mixed with acceptor disk quantitatively labeled with AF594 (**6**)	4.54 ± 0.02	1.5 ± 0.2	9.92
donor disk quantitatively labeled with OG488, coupled to acceptor disk quantitatively labeled with AF594 (**7**)	4.39 ± 0.02	0.86 ± 0.05	35.82

a Values for lifetime components are shown; ± asymptotic standard error, calculated in PicoQuant Fluofit.

By contrast, the short component of the fluorescence lifetime of the donor dye showed more variation according to its protein and chromophore environment. The short component of the fluorescence lifetime of quantitatively labeled assemblies was always decreased when compared to singly labeled assemblies. This indicates that the excited state of OG488 dyes in an array on cpTMV was depleted *via* nonradiative decay from interactions with neighboring dyes (*e.g.*, contact quenching and chromophore aggregation). The most significant decrease in the fluorescence lifetime short component occurred when a donor-labeled disk was oxidatively coupled to an acceptor disk labeled with AF594, shortening the short component of the lifetime from 1.5 ± 0.2 ns in mixed assemblies to 0.86 ± 0.05 ns in coupled assemblies. The short component also had the greatest %amplitude contribution to the fluorescence lifetime in the sample containing coupled donor and acceptor disks. This decrease in the fluorescence lifetime demonstrates that energy transfer occurred between the asymmetrically coupled assemblies from an array of donor to acceptor chromophores.

The fluorescence decay profile was also used to estimate a timescale for energy transfer from donor to acceptor cpTMV disks in the conjugated system (**7**). Based on structural inspection, we estimate the closest interchromophore distance between a donor and acceptor pair to be 7.5 nm. This distance is based on the distance between residue S23C (the chromophore attachment site) on two closely joined disks in a parallel orientation (red line in Figure S17a). However, due to the presence of multiple donor–acceptor pairs and distances present in the coupled disk system, there are multiple donor–acceptor pairs that may participate in energy transfer in a single coupled disk system, with 34 chromophores per disk and a distance distribution between ∼7.5 nm for the closest pair and ∼29 nm for the furthest pair. These distances also do not account for the flexibility afforded by the linkers between the chromophore and the protein, which allow for some translational and rotational flexibility. Considering only the 1/*R*^6^ relationship between interchromophore distance *R* and timescale of transfer τ_T_, there are many donor–acceptor pairs that likely significantly contribute to energy transfer in this model. A subset of the 25 nearest neighbor distances and the proportion each would be expected to contribute to τ_T_ based only on the estimated interchromophore distance *R* are shown in Figure S17a,b. This clearly shows that no single pair of donor and acceptor is expected to dominate energy transfer. No single pathway is expected to contribute greater than 12% to the total, and energy transfer is expected to occur *via* multiple pathways in the absence of information about the chromophores’ dipole orientations. Thus, energy transfer calculations should be taken to indicate transfer between disks rather than between specific pairs of donor and acceptor chromophores.^43,44^

Using the experimentally derived fluorescence lifetimes, the timescale of energy transfer between disks τ_T_ can be estimated at 15 ns, with a corresponding rate of energy transfer *k*_T_ of 0.066 ns^–1^ (see Experimental Section for calculation details). The efficiency *E* was calculated at 21%, indicating that approximately 21% of the excitonic energy was transferred between the donor and acceptor disks in this system. Despite the large interchromophore distances present in this model, the multiple redundant donor–acceptor pathways likely contributed to the observed efficiency.^44^ Energy transfer between LH2 and LH1 in photosynthetic membranes has been found to occur at much faster rates of 3–5 ps.^8^ Alongside a lower spectral overlap and extinction coefficient in our donor–acceptor pairs than in the closely coupled chromophores found in bacterial LH2 and LH1, the primary limiting factor in our model for achieving these rapid rates is the large distance between donor and acceptor chromophores, which at ∼7.5 nm is greater than twice the minimum separation expected between LH2 and LH1 in a photosynthetic membrane.^45^ This distance may be tuned in the future to achieve more rapid energy transfer using chromophore modification sites closer to the disk peripheries;^29^ for example, given the 1/*R*^6^ relationship between interchromophore distance *R* and rate of transfer τ_T_ between FRET chromophore pairs, a decrease in distance from 7.5 to ∼3 nm could result in greater than a 200-fold decrease in τ_T_. Despite the larger interchromophore distances, our model may increase energy transfer efficiency through the presence of multiple inter-ring donor–acceptor energy transfer pathways, a feature which also enhances energy transfer efficiency between LH2 and LH1.^46^

In addition, the close association of the photosynthetic pigments with the protein scaffold of LH1 and LH2 restricts the chromophores’ relative orientational and translational flexibility and contributes to their efficient energy transfer.^4,5^ By contrast, the cpTMV surface modification site used herein positions chromophores on the disks’ surface, which is helpful for achieving high modification levels to avoid gaps in the chromophore arrays but allows for orientational flexibility and limits direct protein–chromophore interactions.^28,29^ Attaching chromophores to the cpTMV surface using more rigid linkers or a modification site in the cavity region between disks as done previously^28^ would allow for the influence of chromophore constraint and chromophore–protein coupling on interassembly energy transfer to be investigated.^28,29^

## Conclusions

We have engineered a protein-based system for simulating energy transfer between photosynthetic light-harvesting components. This tailorable platform mimics the photosynthetic environment in pigment orientation and positioning as well as providing the opportunity to study the effect of chromophore–protein interactions on energy transfer between light-harvesting complexes. In order to construct this system, a method was developed for asymmetric, site-selective protein–protein conjugation employing noncanonical amino acids. Using this method, we were able to achieve site-selective labeling of protein complexes with a dye at a specific engineered cysteine residue and to an adjacent protein complex at the engineered noncanonical amino acid residues *p*AF and 3NY. This model system demonstrated energy transfer from an array of donor chromophores to an array of acceptor chromophores in a well-ordered conformation and solvent environment at an efficiency of 21%. Due to the versatility of the system, this efficiency may be improved by changing the protein modification site, dye linker composition, or dye identities to improve donor–acceptor spectral overlap and decrease donor–acceptor distances.

The installation of noncanonical amino acids into proteins *via* protein engineering and amber codon suppression is becoming increasingly accessible.^47,48^ The protein–protein conjugation developed herein is the first example of a protein containing an engineered *p*AF residue being directly and asymmetrically coupled to a protein containing an engineered 3NY residue, adding to the library of protein–protein conjugation strategies. This method may therefore be useful for designing both synthetic light-harvesting systems of increasing complexity and other protein-based materials, such as well-controlled antibody–protein conjugates.

In a recent report, we have demonstrated the ability to incorporate the TMV-based light-harvesting model into a supported lipid bilayer.^49^ We have also recently used the TMV model to identify sources of disorder in biomimetic light-harvesting systems and their effect on long-range energy transfer.^29^ The ability to couple two TMV assemblies asymmetrically adds another layer of utility to the TMV-based model system by allowing energy transfer between donor and acceptor complexes at controlled distances and orientations to be examined.

## Experimental Section

### General Methods

Unless otherwise noted, all chemicals and solvents were of analytical grade and received from commercial sources. Water (dd-H_2_O) used in biological procedures and as a reaction solvent was deionized using a Barnstead NANOpure purification system (ThermoFisher, Waltham, MA). Oligonucleotides were purchased from Integrated DNA Technologies (Coralville, IA) and MilliporeSigma (Burlington, MA). The pDule-*para*-aminoPhe (Addgene #85502),^36^ pDule-3-nitroTyrosine (Addgene #85498),^50^ and pBad-sfGFP 150TAG (Addgene #85483)^51^ plasmids were gifts from Ryan Mehl. Amicon Ultra MWCO centrifugal concentrators were obtained from MilliporeSigma (St. Louis, MO).

### General Instrumentation and Sample Analysis

UV–vis absorption measurements were conducted on a Cary UV–vis 100 spectrophotometer (Agilent, USA). Protein concentration was determined by UV–vis analysis on a Nanodrop 1000 instrument (Nanodrop, USA) by monitoring absorbance at 280 nm. Electrospray LC/MS analysis of proteins and their bioconjugates was performed using an Agilent 1200 series liquid chromatograph (Agilent Technologies, USA) that was connected in-line with an Agilent 6224 time-of-flight (TOF) LC/MS system equipped with a Turbospray ion source. Protein samples were run with a Proswift RP-4H column (Dionex, USA). Protein mass reconstruction was performed on the charge ladder with Mass Hunter software (Agilent, USA). High-performance liquid chromatography (HPLC) was performed on Agilent 1200 Series HPLC Systems (Agilent, USA). Sample analysis for all HPLC experiments was achieved with an in-line diode array detector and in-line fluorescence detector. SEC was performed using a Polysep-GFC-P-5000 column (4.6 × 250 mm) (Phenomenex, USA) at 1.0 mL/min using a mobile phase of 10 mM sodium phosphate buffer, pH 7.2. DLS was performed on a Zetasizer Nano Series (Malvern Instruments, UK). Measurements were taken in triplicate at protein concentrations of 0.2–1.0 mg/mL in 10 mM sodium phosphate buffer, pH 7.2, at 25 °C.

### Gel Analyses

Sodium dodecyl sulfate-polyacrylamide gel electrophoresis (SDS-PAGE) was carried out in a Mini cell tank apparatus (Life Technologies, Carlsbad, CA) using NuPAGE Novex 4–12% Bis-Tris Protein Gels (Life Technologies). The sample and electrode buffers were prepared according to the suggestions of the manufacturer. All protein electrophoresis samples were heated for 5–10 min at 95 °C in the presence of 1,4-dithiothreitol (DTT) to ensure the reduction of disulfide bonds. Gels were run for 30 min at 200 V to separate the bands. Commercially available markers (Bio-Rad) were applied to at least one lane of each gel for the assignment of apparent molecular masses. Native agarose gel electrophoresis was performed using 0.9% agarose gels and 50 mM sodium phosphate buffer, pH 7.2. Samples were mixed with 80% glycerol at a sample/glycerol ratio of 1:1 and allowed to settle in wells for 10 min prior to applying voltage. Native gels were placed on ice and run for 8 h at 25 V to separate the bands. Visualization of protein bands was accomplished by staining with Coomassie Brilliant Blue R-250 (Bio-Rad, Hercules, CA). Gel imaging was performed on a Gel Doc (Bio-Rad, Hercules, CA).

### Transmission Electron Microscopy

Samples were prepared for TEM analysis using negative staining. Analyte solution (0.2–1 mg/mL cpTMV in 10 mM sodium phosphate buffer, pH 7.2) was applied to carbon-coated copper grids for 2 min, followed by rinsing in 4 × 10 μL droplets of a 1% aqueous solution of uranyl acetate. Grids were left in the final droplet for 1 min. TEM images were obtained at the Berkeley Electron Microscope Lab using an FEI Tecnai 12 transmission electron microscope with an accelerating voltage of 100 kV.

### Protein Expression and Purification

The production of *p*AF- and 3NY-containing proteins was performed according to previously published protocols.^38,39^ Briefly, the pBAD-cpTMV-S65* vectors (with * designating the stop codon TAG) with either pDule-*para*-aminoPhe or pDule-3-nitroTyrosine were cotransformed into DH10B *E. coli* cells and plated on LB agar plates containing 50 μg/mL ampicillin and 12.5 μg/mL tetracycline. The resulting colonies were grown overnight in 10 mL of LB containing 50 μg/mL ampicillin and 12.5 μg/mL tetracycline at 37 °C and then added to 1 L of arabinose auto-induction media. The 1 L culture was allowed to shake at 37 °C, 220 rpm until it reached an OD_600_ of 0.6–0.8. Then, 10 mL of a 100 mM solution of the noncanonical amino acid (*p*AF or 3NY) was added to the growth medium to a final concentration of 1 mM. The culture was incubated at 37 °C, 220 rpm for an additional 18 h. Cell pellets were collected at 8000 rpm for 30 min, after which the supernatant was discarded and the cell pellets were frozen at −20 °C until purification. After freezing, cell pellets were partially thawed and resuspended in 10 mL of lysis buffer [20 mM triethanolamine (TEA), pH 7.2]. Cells were lysed by sonication with a 2 s on and 4 s off cycle for a total of 10 min using a standard disruptor horn at an amplitude of 60% (Branson Ultrasonics, Danbury, CT). The resulting lysate was cleared at 14,000 rpm for 30 min. The supernatant was treated with 30–40% volume (3–4 mL) of saturated ammonium sulfate and allowed to rotate for 10 min at 4 °C to allow for complete protein precipitation. The precipitated protein was collected at 11,000 rpm for 30 min and resuspended in 10 mL of lysis buffer. Complete redissolving of the protein and removal of residual ammonium sulfate was accomplished by performing dialysis in 1 L of lysis buffer overnight with at least one buffer exchange. The resulting protein solution was treated with 5 μL of benzonase (MilliporeSigma, St. Louis, MO) and 4 mg of MgCl_2_ at room temperature for 30 min before the solution was spun down at 10,000 rpm for 10 min. The resulting supernatant was filtered through a 0.22 μm filter and purified using a DEAE column with a 0–180 mM NaCl gradient elution in 20 mM TEA buffer, pH 7.2. The fractions containing cpTMV were further purified using a Sephacryl S-500 column in 10 mM sodium phosphate elution buffer (pH 7.2). Pure fractions were collected and concentrated using 100 kDa MWCO centrifugal concentrators. Purity was confirmed by SDS-PAGE and ESI-TOF MS. Assembly state was confirmed by HPLC-SEC, DLS, and TEM. The protein was flash-frozen and stored indefinitely at −80 °C or stored for no longer than 2 weeks at 4 °C without observed decomposition or change in the assembly state.

### Screen of Protein Expression Conditions

The pBAD-cpTMV vectors containing the relevant noncanonical amino acid amber codon (or pBAD-sfGFP 150TAG for GFP controls) were cotransformed with pDule-3-nitroTyrosine into DH10B *E. coli* cells and plated on LB agar plates containing 50 μg/mL ampicillin and 12.5 μg/mL tetracycline. The resulting colonies were grown overnight in 10 mL of LB containing 50 μg/mL ampicillin and 12.5 μg/mL tetracycline at 37 °C and then 25 μL of the overnight culture was added to 5 mL of arabinose auto-induction media^38^ with modifications, as shown in Figure S1. The cultures were then shaken at 37 °C, 220 rpm. At the times specified in Figure S1, 250 μL of a 20 mM solution of the noncanonical amino acid (3NY) was added to the growth medium to a final concentration of 1 mM. The culture was incubated at 37 °C, 220 rpm for a total of 23 h. 500 μL of culture from each expression was then centrifuged at 13,200 rpm for 5 min. The supernatant was discarded, and the pellet was resuspended in 250 μL of Bugbuster Protein Extraction Reagent (MilliporeSigma, St. Louis, MO) and allowed to sit at room temperature for 20 min. The solution was then centrifuged for 20 min at 13,200 rpm, the supernatant was removed and retained, and the insoluble portion was resuspended in lysis buffer. The expression level in the supernatant and resuspended insoluble portions were analyzed using gel electrophoresis.

### General Procedure for Labeling cpTMV Thiols with Maleimide Dyes

The following procedure is based on a previously reported procedure with minimal modifications.^28^ To 100 μL of cpTMV (100 μM in 10 mM sodium phosphate buffer, pH 7.2) was added 5 equiv of TCEP to ensure complete thiol reduction prior to chromophore modification. The mixture was briefly vortexed and allowed to sit at room temperature for 20 min. Excess TCEP was removed with a NAP-5 Sephadex G-25 column (GE Healthcare, USA), followed by spin concentration of the eluent in 100 kDa MWCO cutoff filters. To 100 μL of the reduced cpTMV (100 μM in 10 mM sodium phosphate buffer, pH 7.2) was added 0.01 equiv of maleimide-functionalized chromophore for single modification or 5 equiv of functionalized chromophore for full modification. The reaction mixture was briefly vortexed and then incubated in 1.5 mL Eppendorf tubes at room temperature with an aluminum foil cover. After 2 h, the crude reactions were purified with a NAP-5 Sephadex G-25 column, followed by spin concentration of the eluent in 100 kDa MWCO concentrators, and an additional NAP-5 Sephadex G-25 column to remove excess chromophores. Further spin concentration was achieved using 100 kDa MWCO concentrators. The protein conjugates were analyzed with MS to gauge the extent of modification and with HPLC-SEC for assessment of purity and validation of the assembly state. Protein conjugates were wrapped in foil and stored at 4 °C for several days or flash-frozen and stored at −80 °C for longer periods.

### General Procedure for Labeling *p*AF-Containing cpTMV with Aminophenols or Catechols

To 96 μL of 20 μM *p*AF-containing cpTMV in 100 mM sodium phosphate buffer (pH 6.5) were added 5 equiv of amino-*p*-cresol or 4-methylcatechol (2 μL of 5 mM aminophenol or catechol in 1:10 acetonitrile/100 mM sodium phosphate buffer, pH 6.5) and 5 equiv of potassium ferricyanide (2 μL of 5 mM potassium ferricyanide in 100 mM sodium phosphate buffer, pH 6.5). The reaction was briefly vortexed and incubated at room temperature. After 2 h, the crude reactions were purified with a NAP-5 Sephadex G-25 column, followed by spin concentration of the eluent in 100 kDa MWCO cutoff filters. The protein conjugates were analyzed with MS to gauge the extent of modification.

### General Procedure for Reduction of 3NY-Containing cpTMV

To 490 μL of 20 μM 3NY-containing cpTMV in 100 mM sodium phosphate buffer (pH 6.5) was added 200 equiv of sodium dithionite [10 μL of 200 mM sodium dithionite in 100 mM sodium phosphate buffer (pH 6.5) always prepared immediately prior to use]. 800 equiv of sodium dithionite was used for dye-labeled, 3NY-containing cpTMV. The mixture was briefly vortexed and incubated at room temperature. After 40 min (or 2 h for dye-labeled, 3NY-containing cpTMV), the crude reactions were purified with a NAP-5 Sephadex G-25 column, followed by spin concentration of the eluent in 100 kDa MWCO cutoff filters, and an additional NAP-5 Sephadex G-25 column to remove the excess reducing agent. The protein conjugates were analyzed with MS to gauge the extent of reduction and with HPLC-SEC for assessment of purity and validation of the assembly state.

### General Procedure for Labeling 3AY-Containing cpTMV with *p*-Toluidine

To 96 μL of 20 μM 3AY-containing cpTMV in 100 mM sodium phosphate buffer (pH 6.5) were added 5 equiv of *p*-toluidine (2 μL of 5 mM *p*-toluidine in 1:10 acetonitrile/100 mM sodium phosphate buffer, pH 6.5) and 5 equiv of potassium ferricyanide (2 μL of 5 mM potassium ferricyanide in 100 mM sodium phosphate buffer, pH 6.5). The reaction was briefly vortexed and incubated at room temperature. After 2 h, the crude reactions were purified with a NAP-5 Sephadex G-25 column, followed by spin concentration of the eluent in 100 kDa MWCO cutoff filters. The protein conjugates were analyzed with MS to gauge the extent of modification.

### Procedure for Oxidatively Coupling cpTMV-S65-*p*AF to cpTMV-S65-3AY

To 98 μL of 20 μM *p*AF-containing cpTMV and 10 μM 3AY-containing cpTMV in 100 mM sodium phosphate buffer (pH 6.5) was added 5 equiv of potassium ferricyanide (2 μL of 5 mM potassium ferricyanide in 100 mM sodium phosphate buffer, pH 6.5). The reaction was briefly vortexed and incubated at room temperature. After 2 h, the crude reactions were purified with a NAP-5 Sephadex G-25 column, followed by spin concentration of the eluent in 100 kDa MWCO cutoff filters. Separation of the coupled product from the uncoupled starting material was performed using a Polysep-GFC-P-5000 column (4.6 × 250 mm). The protein conjugates were analyzed with MS to gauge the extent of modification and with HPLC-SEC, DLS, and TEM for assessment of the assembly state.

### Spectroscopic Measurements

Samples in buffer were diluted to 1.5 μM Oregon Green 488 using UV–vis absorption prior to fluorescence measurements. Fluorescence emission and lifetime spectra were collected using a PicoQuant FluoTime FT-300 fluorometer. The samples were transferred to a 1 cm path-length quartz cuvette and excited with a 465 nm PicoQuant pulsed diode laser, with an instrument response function of 150 ps, as measured with a scattering LUDOX sample. Time-resolved emission measurements were performed *via* time-correlated single photon counting (TCSPC). The lifetime values result from mono- or biexponential reconvolution fitting using PicoQuant FluoFit software version 4.6.6.0, with χ^2^ < 1.1 for all measurements.

### FRET and Efficiency Calculations

The interchromophore distances in this system are expected to be over 7 nm for donor/acceptor pairs and ∼1.95 nm for identical chromophores within a single cpTMV disk. Due to these distances, any energy transfer between chromophores in this system would be due to FRET. To calculate the rate of energy transfer between donor and acceptor disks, the expression
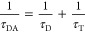
was used, where τ_DA_ is the fluorescence lifetime of the donor disks when coupled to acceptor disks, τ_D_ is the fluorescence lifetime of the donor disks in the absence of acceptor disks, and τ_T_ is the timescale of energy transfer between donors and acceptors.^52^ The rate of energy transfer *k*_T_ is the inverse of τ_T_
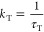


To calculate the efficiency of energy transfer between donor and acceptor disks, the following expression was used
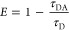
where *E* indicates energy transfer efficiency.^53^

Interchromophore distances were measured using the tape measure tool in ChimeraX, version 1.2.5. To calculate the proportion that each donor–acceptor pair shown in Figure S17a would be expected to contribute to energy transfer based solely on interchromophore distance, the following expression was used
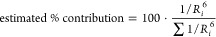
where *R*_*i*_ is the distance between a single donor–acceptor pair.

## Supporting Information Available

SDS-PAGE and native gels, TEM images, MS data, SEC traces, static and time-resolved fluorescence emission spectra, and estimated pigment pair distances and their energy transfer contributions (PDF)
