# Supplementary material for: Protein-Based Model for Energy Transfer between Photosynthetic Light-Harvesting Complexes Is Constructed Using a Direct Protein–Protein Conjugation Strategy
Source: J Am Chem Soc. 2023 Jul 13;145(29):15827–37. doi: 10.1021/jacs.3c02577 (PMC10375525; doi:10.1021/jacs.3c02577)
Supplement: Supplementary file 1 — ja3c02577_si_001.pdf [file ja3c02577_si_001.pdf]

Supporting Information

Protein-Based Model for Energy Transfer between Photosynthetic Light Harvesting Complexes Is Constructed Using a Direct Protein–Protein Conjugation Strategy

Amanda J. Bischoff,[1,2] Leo M. Hamerlynck,[1,2] Amanda J. Li,[1] Trevor D. Roberts,[1] Naomi S. Ginsberg,[1,2,3,4,5] Matthew B. Francis*,[1,2]

[1]Department of Chemistry, University of California, Berkeley, California, 94720, United States

[2]Molecular Biophysics and Integrated Bioimaging Division, Lawrence Berkeley National Laboratory, Berkeley, California, 94720, United States

[3]Department of Physics, University of California, Berkeley, California, 94720, United States

[4]Kavli Energy NanoScience Institute, Berkeley, California, 94720, United States

[5]Materials Sciences Division, Lawrence Berkeley National Laboratory, Berkeley, California, 94720, United States

Table of contents

Figure S1. Screen of peripheral sites for expression of cpTMV containing the noncanonical amino acid 3-nitrotyrosine (3NY).	2
Figure S2. Assembly state of cpTMV-S65-3NY.	3
Figure S3. Reduction of cpTMV-S65-3NY.	4
Figure S4. Verification of the cpTMV modification site during oxidative coupling using small molecules and cpTMV-S23 as a control.	5
Figure S5. Evidence for protein–protein coupling of cpTMV assemblies containing noncanonical amino acids.	6
Figure S6. TEM images of coupled and mixed assemblies.	7
Figure S7. Masses of expressed proteins and protein–dye conjugates.	8
Figure S8. Characterization of protein–dye and protein–protein conjugates for energy transfer experiments.	9
Figure S9. Emission spectra of the samples shown in Figure 4c and a sample without a donor-labeled disk.	10
Figure S10. Fluorescence emission over time of sample **1**.	11
Figure S11. Fluorescence emission over time of sample **2**.	12
Figure S12. Fluorescence emission over time of sample **3**.	13
Figure S13. Fluorescence emission over time of sample **4**.	14
Figure S14. Fluorescence emission over time of sample **5**.	15
Figure S15. Fluorescence emission over time of sample **6**.	16
Figure S16. Fluorescence emission over time of sample **7**.	17
Figure S17. An estimate of the contributions of different donor–acceptor chromophore pairs in sample **7** to inter-disk energy transfer.	18

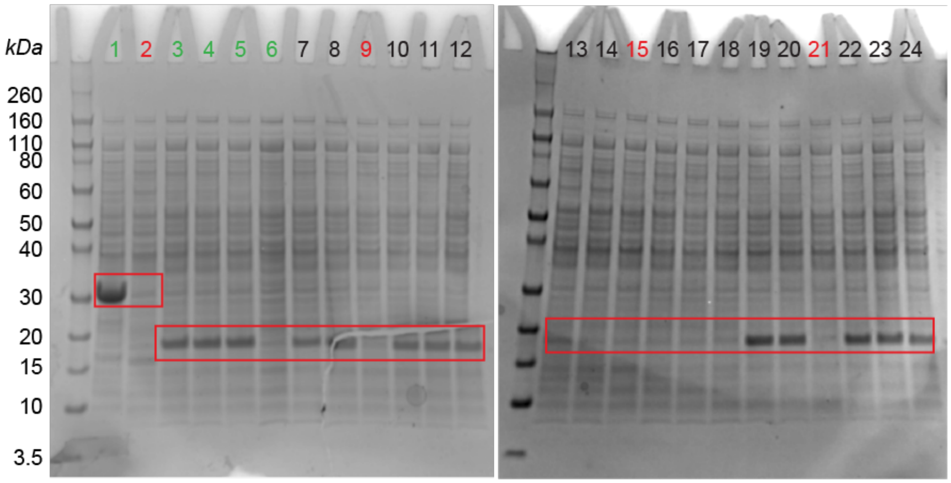

lane	protein	mutation	arabinose concentration (% w/v)	3NY addition time (h)	arabinose addition time (h)
1	GFP	N150-3NY	0.05	3.5	0
2	GFP	N150-3NY	0.05	-	0
3	cpTMV	S23C	0.05	3.5	0
4	cpTMV	S23C	0.05	2.5	0
5	cpTMV	S23C	0.05	-	0
6	cpTMV	S23C	0.05	3.5	0.5
7	cpTMV	S63-3NY	0.05	3.5	0
8	cpTMV	S63-3NY	0.05	2.5	0
9	cpTMV	S63-3NY	0.05	-	0
10	cpTMV	S63-3NY	0.05	3.5	0.5
11	cpTMV	S63-3NY	0.1	3.5	0
12	cpTMV	S63-3NY	0.1	3.5	0.5
13	cpTMV	Y64-3NY	0.05	3.5	0
14	cpTMV	Y64-3NY	0.05	2.5	0
15	cpTMV	Y64-3NY	0.05	-	0
16	cpTMV	Y64-3NY	0.05	3.5	0.5
17	cpTMV	Y64-3NY	0.1	3.5	0
18	cpTMV	Y64-3NY	0.1	3.5	0.5
19	cpTMV	S65-3NY	0.05	3.5	0
20	cpTMV	S65-3NY	0.05	2.5	0
21	cpTMV	S65-3NY	0.05	-	0
22	cpTMV	S65-3NY	0.05	3.5	0.5
23	cpTMV	S65-3NY	0.1	3.5	0
24	cpTMV	S65-3NY	0.1	3.5	0.5

Figure S1. Screen of peripheral sites for expression of cpTMV containing noncanonical amino acid 3-nitrotyrosine (3NY). An SDS-PAGE gel of the soluble fraction of small-scale expressions with the variations shown in the corresponding table shows that the S65-3NY mutant was expressed at the highest level of mutants screened. Positive controls (GFP-N150-3NY and cpTMV-S23C without a noncanonical amino acid) and their expression conditions are shown in green. Negative controls to which no 3NY was added are shown in red. Red outlines superimposed on the SDS-PAGE gels indicate the expected MW for GFP (28 kDa) and cpTMV (17 kDa).

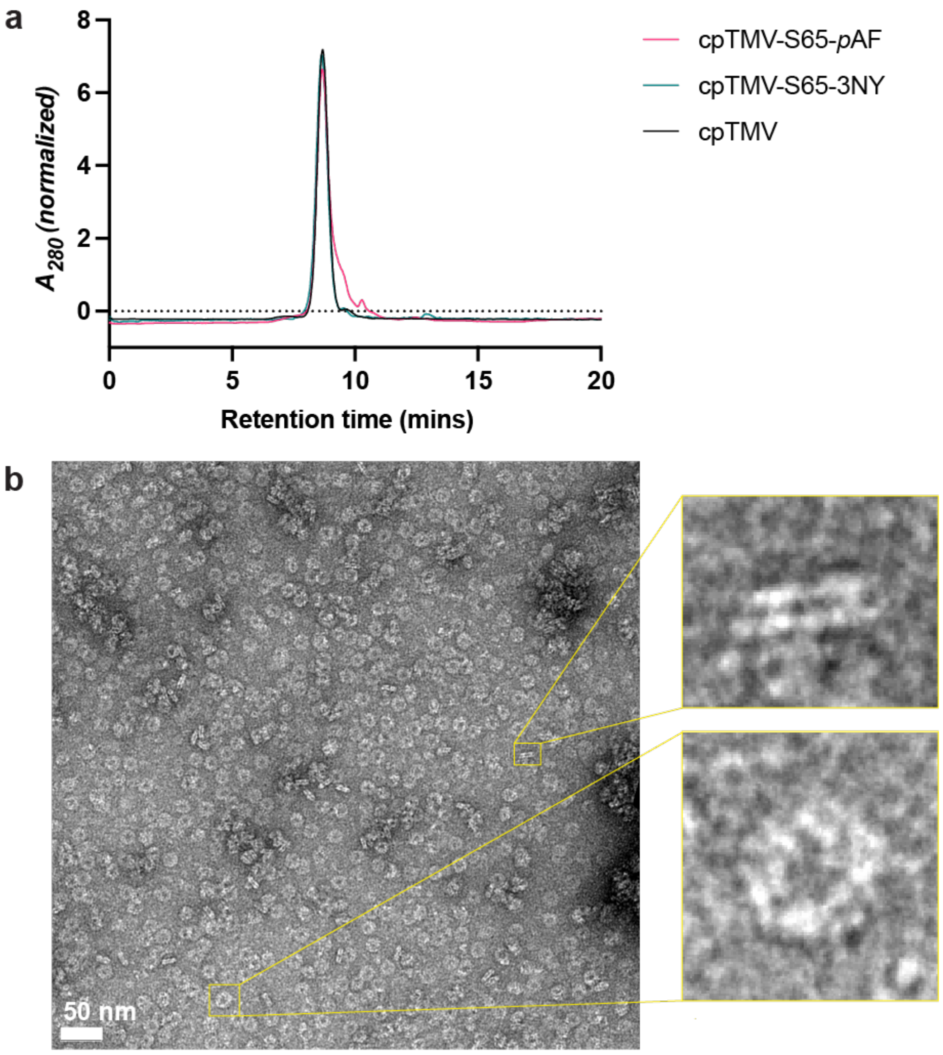

Figure S2. Assembly state of cpTMV-S65-3NY. (a) The size of both cpTMV-S65-*p*AF and cpTMV-S65-3NY matches that of previously reported cpTMV as demonstrated by size exclusion chromatography. (b) Transmission electron microscopy (TEM) images of cpTMV-S65-3NY demonstrate that it assembles into double-layered disks similar to other cpTMV mutants. Enlarged images show a disk on its side with two layers (top image) and on its face with a central pore (bottom image).

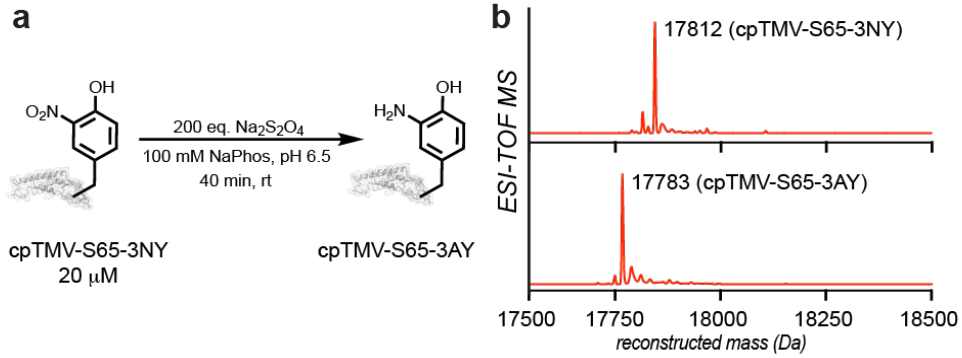

Figure S3. Reduction of cpTMV-S65-3NY. (a) cpTMV-S65-3NY is reduced to cpTMV-S65-3AY (aminotyrosine) using sodium dithionite. (b) The peak area integration ratio of masses of the protein upon reduction indicates that cpTMV-S65-3AY was formed at 83% yield.

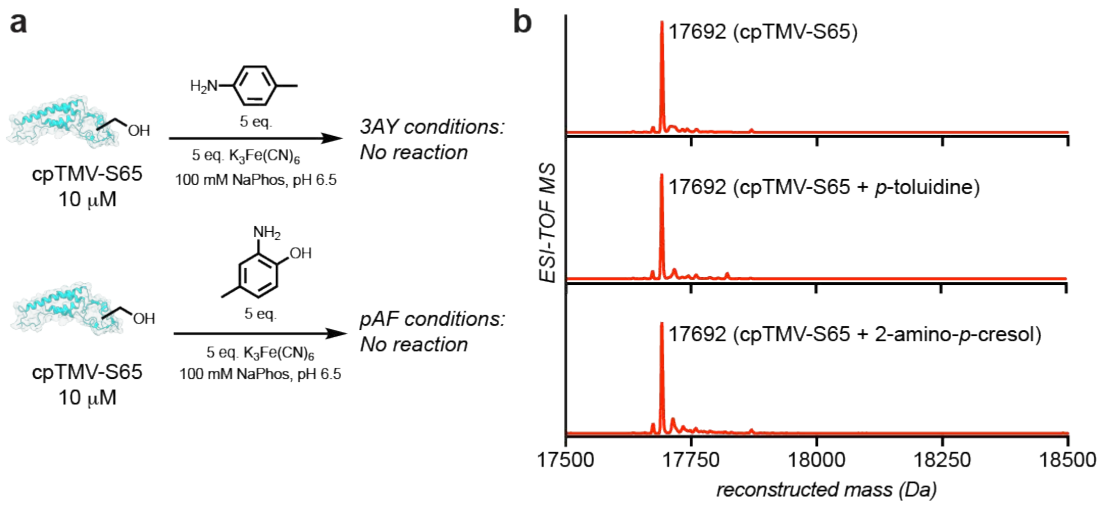

Figure S4. Verification of the cpTMV modification site during oxidative coupling using small molecules and cpTMV-S65 as a control. (a) Reaction schemes show control reactions using cpTMV-S65 incubated with *p*-toluidine (3AY conditions) or 2-amino-*p*-cresol (*p*AF conditions) and potassium ferricyanide. (b) No appreciable difference in the mass of cpTMV-S65 (MW = 17692) was observed after incubation with small molecules as shown in the reaction schemes.

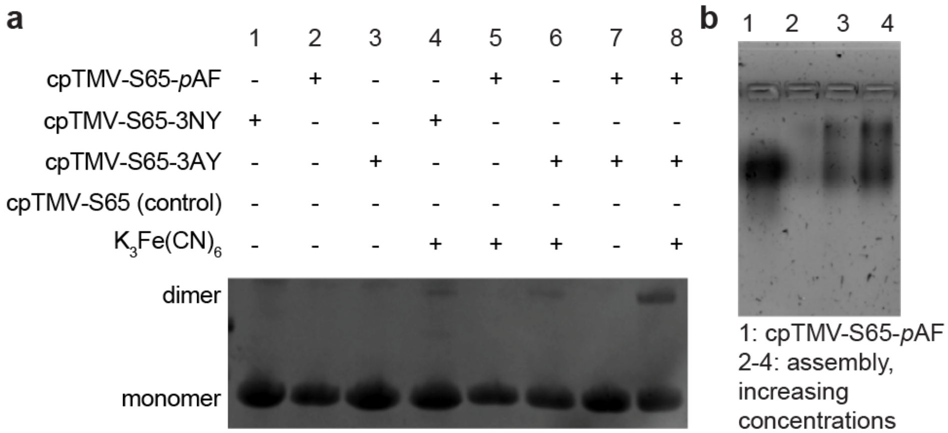

Figure S5. Evidence for protein–protein coupling of cpTMV assemblies containing non-canonical amino acids. (a) An SDS-PAGE gel shows disk coupling and controls. (b) A verification of size increase from individual disks to assemblies using a native agarose gel: 0.9% agarose in 50 mM NaPhos, pH 7.2, 8 h, 0 °C.

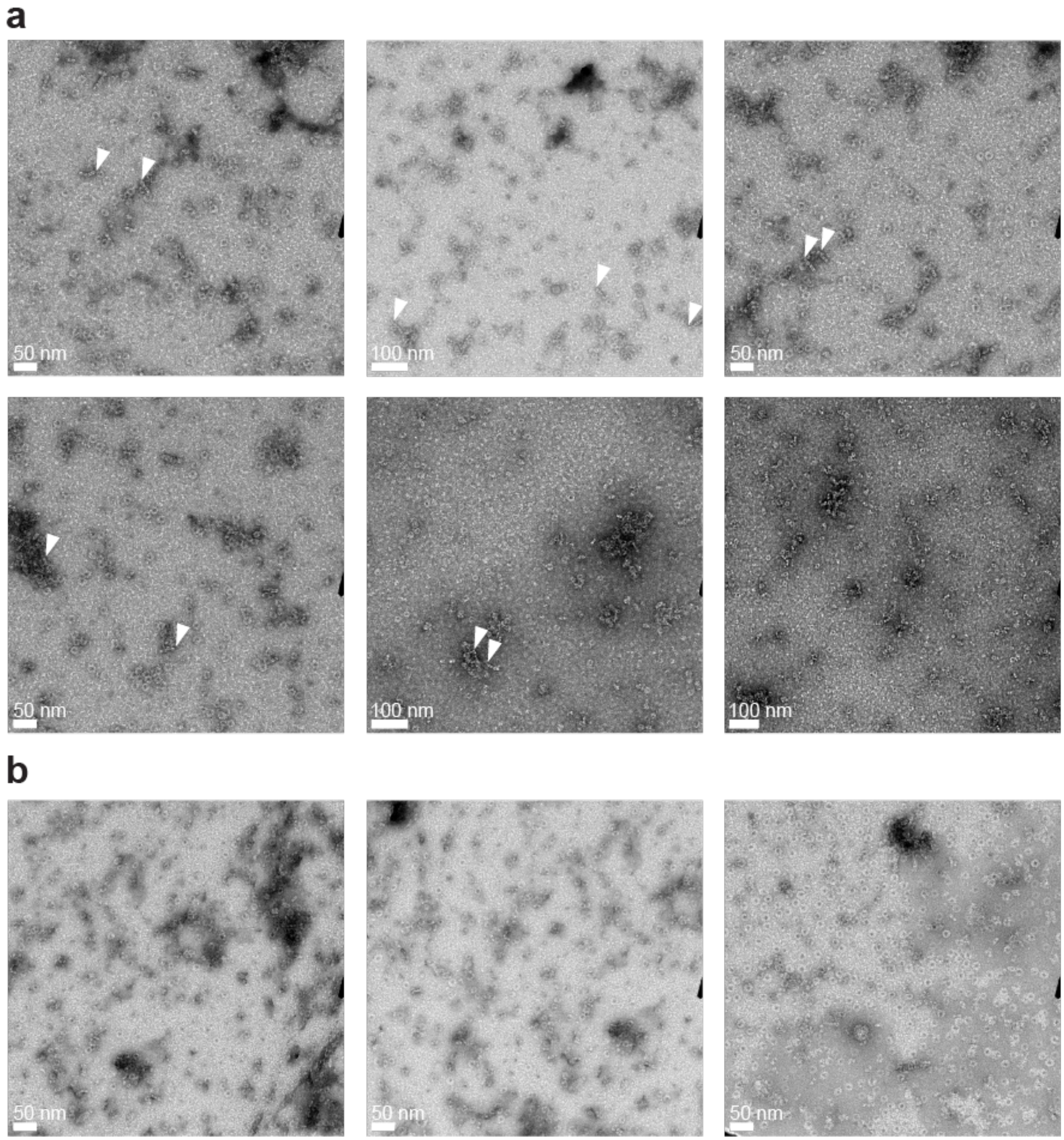

Figure S6. TEM images of coupled and mixed assemblies. (a) TEM images of cpTMV-S65-*p*AF coupled to cpTMV-S65-3AY with potassium ferricyanide show multiple examples of disks that appear conjugated at their peripheries. White arrows indicate images of conjugated cpTMV disks from a side view, with disks at or near a 180° angle to one another. (b) TEM images of cpTMV-S65-*p*AF mixed with cpTMV-S65-3AY in the absence of oxidant show some examples of disks on their faces that are adjacent to one another. Conjugated disks on their sides are not observed.

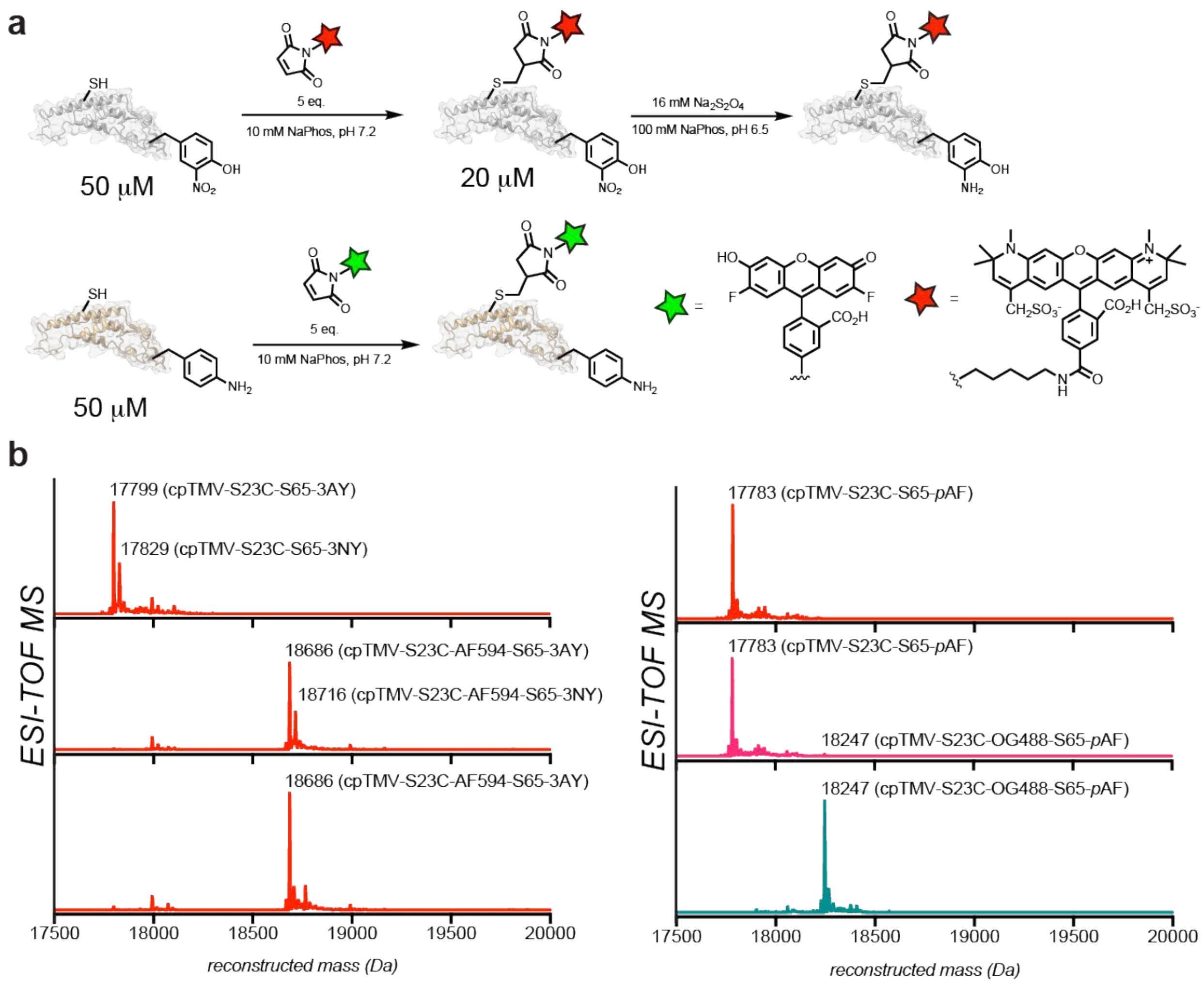

Figure S7. Masses of expressed proteins and protein–dye conjugates. (a) Conditions are shown for the conjugation of maleimide dyes to cpTMV with a cysteine at position 23 and reduction of the noncanonical amino acid 3NY to 3AY. (b) Mass spectra for quantitative labeling of the 3NY-containing cpTMV with Alexa Fluor 594 maleimide (AF594) (expected MW: 18713 Da), reduction of 3NY to 3AY (expected MW: 18683 Da), and single (**2**) and full (**3**) labeling of the *p*AF-containing cpTMV by Oregon Green 488 maleimide (OG488) (expected MW: 18247 Da) are shown. For relevant samples, colors match those shown in Figure 4c.

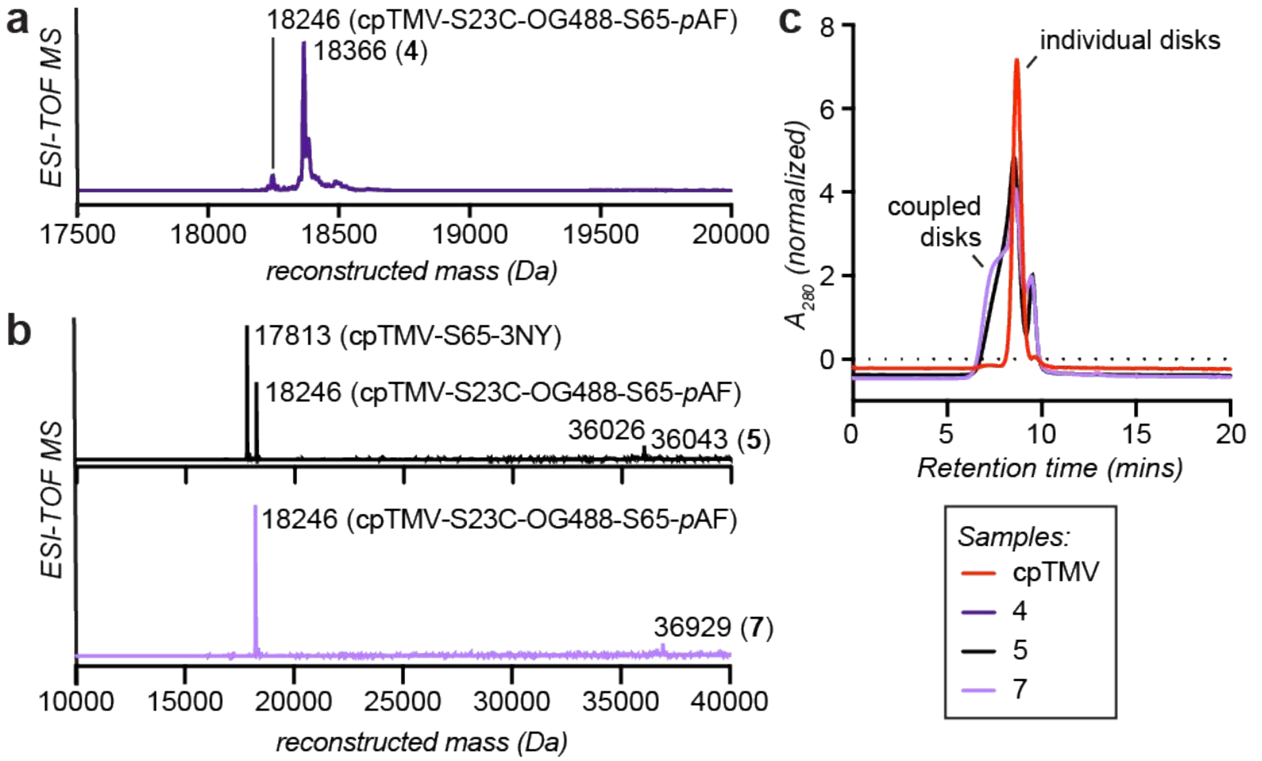

Figure S8. Characterization of protein–dye and protein–protein conjugates for energy transfer experiments. (a) Mass spectrometry data shows cpTMV disks labeled with OG488 and coupled to 4-methylcatechol at the S65 site (**4**) at 91% modification (expected MW of dye-labeled, coupled disks: 18367 Da). (b) Mass spectrometry data shows cpTMV disks fully labeled with OG488 and coupled to cpTMV disks with no chromophore labeling (**5**) (expected MW: 36043 Da) and cpTMV disks fully labeled with OG488 coupled to cpTMV disks fully labeled with AF594 (**7**) (expected MW: 36927 Da). (c) Size-exclusion chromatography shows cpTMV disks fully labeled with OG488 and coupled to cpTMV disks with no chromophore labeling (**5**) and cpTMV disks fully labeled with OG488 coupled to cpTMV disks fully labeled with AF594 (**7**). An uncoupled cpTMV sample forming only individual, double-layered disks is shown for comparison. For relevant samples, colors match those shown in Figure 4c.

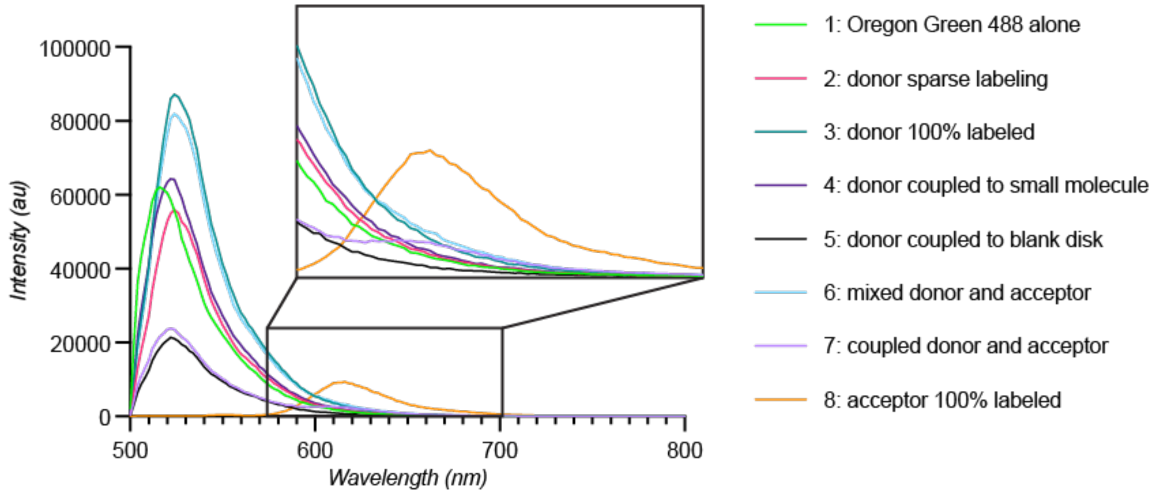

Figure S9. Emission spectra of the samples shown in Figure 4c and a sample without donor-labeled disks. These un-normalized spectra show that in the absence of the donor chromophore OG488, excitation and emission of the acceptor chromophore AF594 conjugated to cpTMV-S65-3NY disks can occur. In the presence of a donor chromophore, such as in the case of mixed disks containing donor and acceptor chromophores, this excitation does not occur.

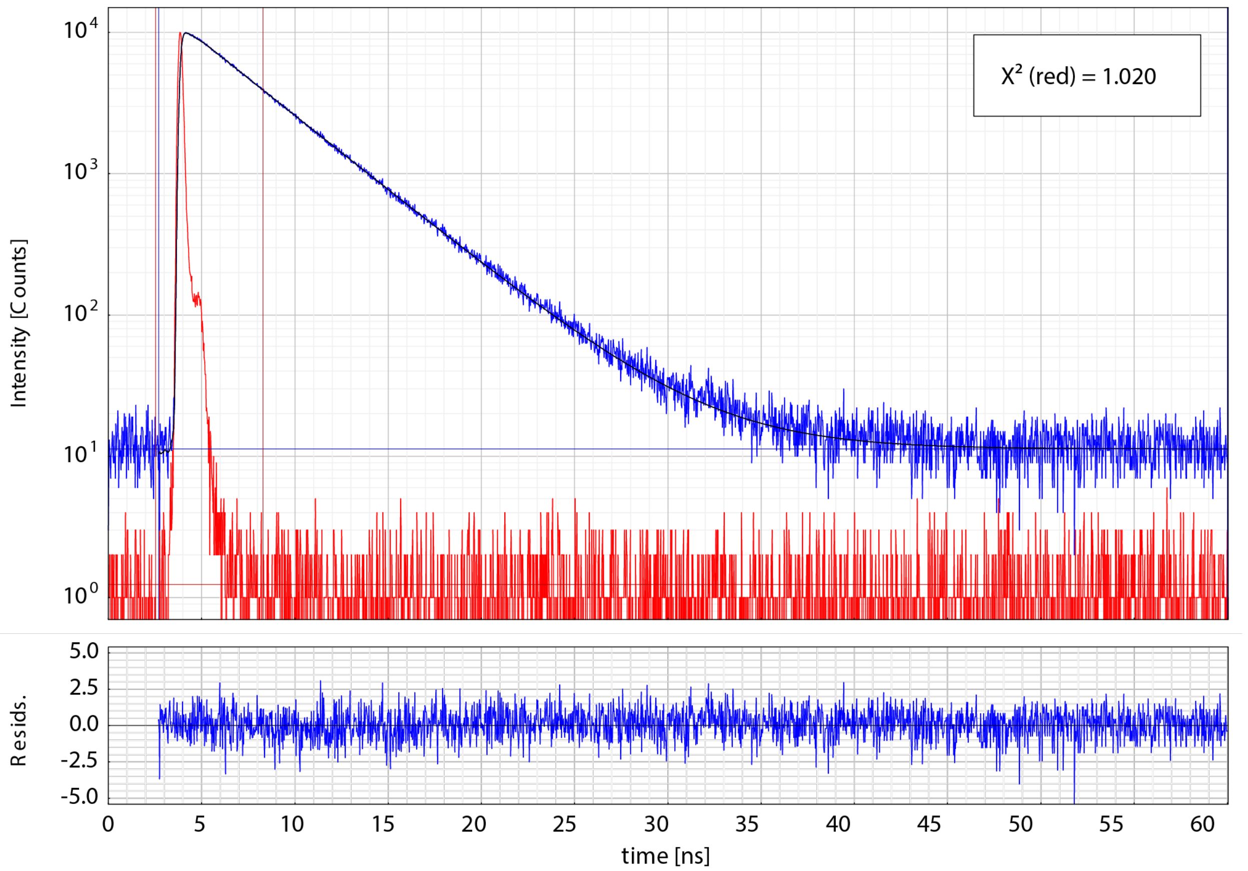

Figure S10. Fluorescence emission over time of sample **1**. Emission at 524 nm after excitation at 465 nm of Oregon Green 488 capped with Mesna (**1**) is shown in blue and the instrument response function in red. The average fluorescence lifetime is well-described by a monoexponential fit as shown by the fit residuals.

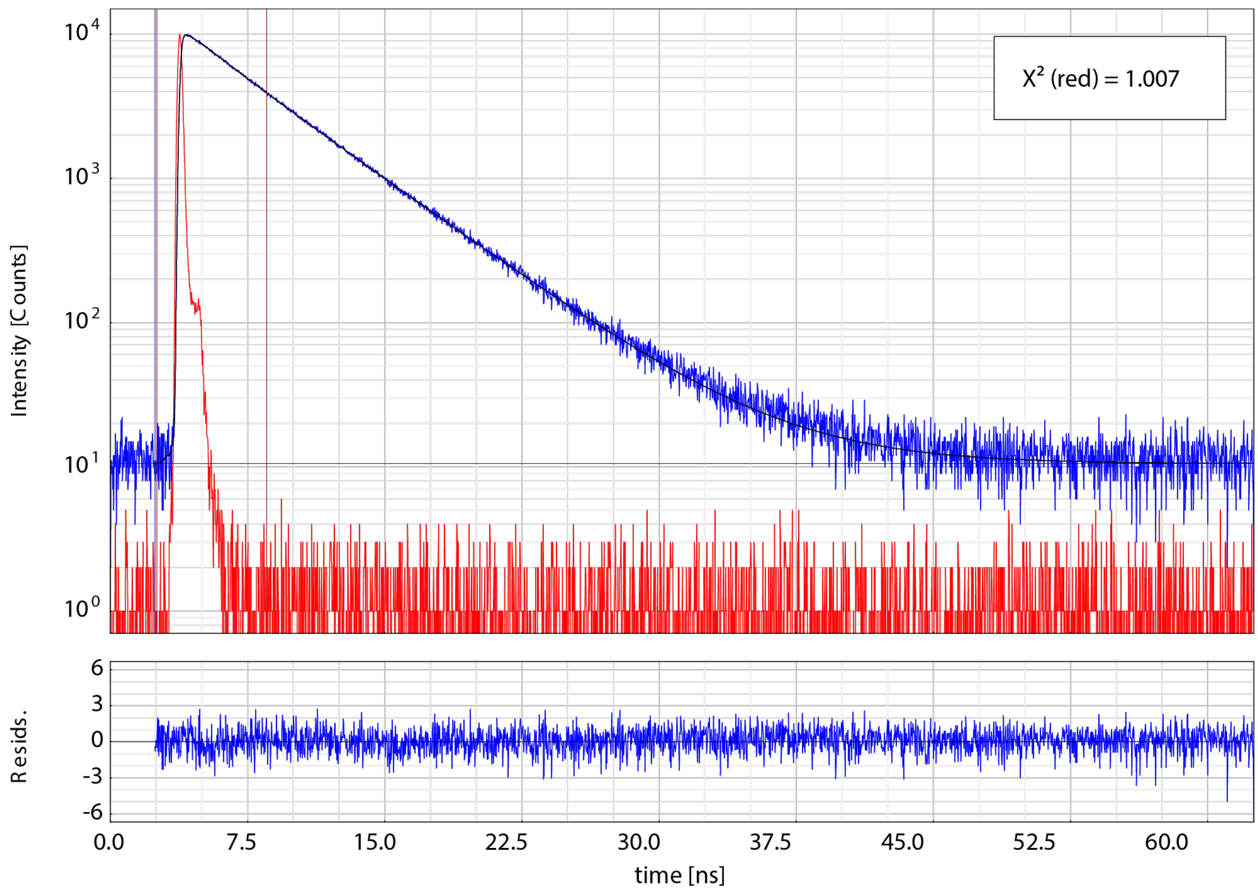

Figure S11. Fluorescence emission over time of sample **2**. Emission at 524 nm after excitation at 465 nm of cpTMV-S23C-S65-*p*AF disk assemblies labeled with Oregon Green 488 at a ratio of one dye per disk (**2**) is shown in blue and the instrument response function in red. The average fluorescence lifetime is well-described by a biexponential fit as shown by the fit residuals.

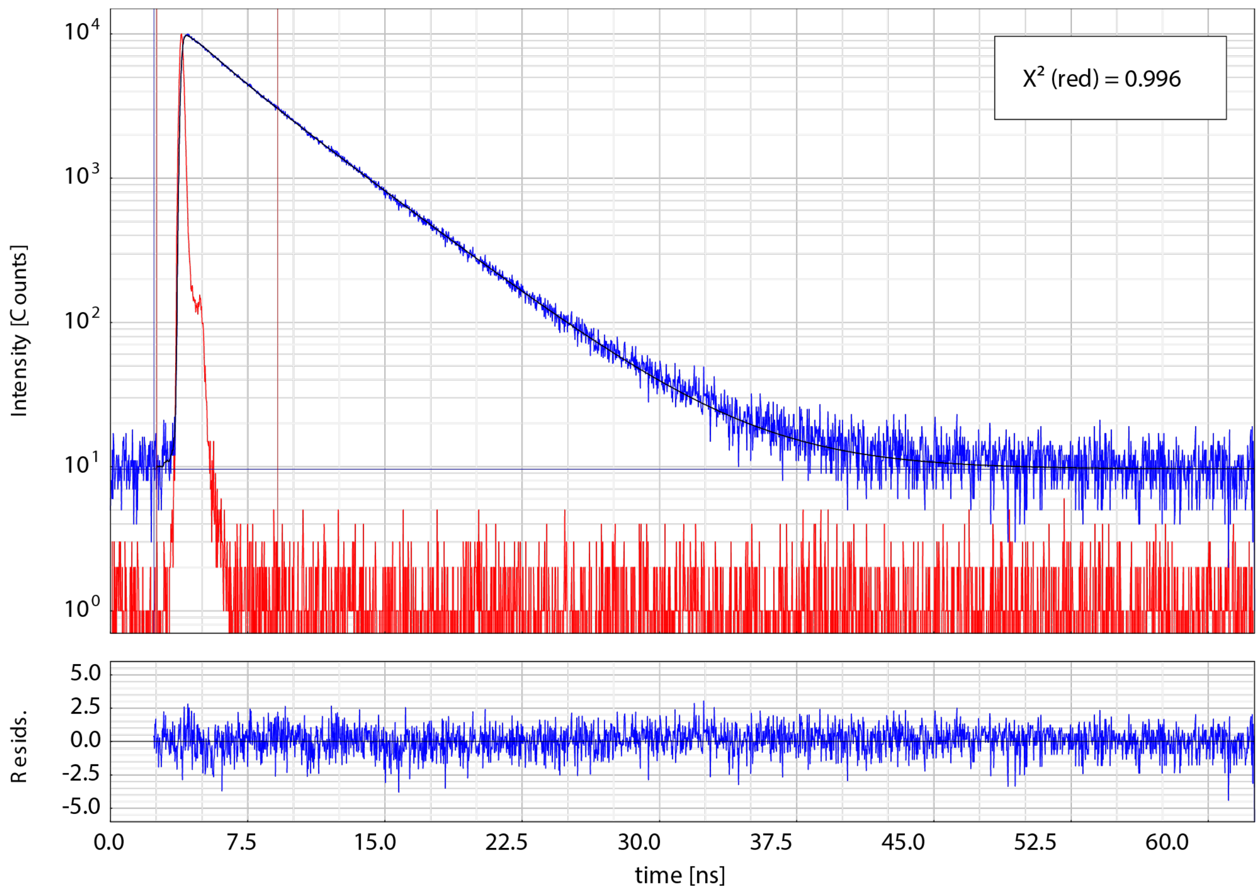

Figure S12. Fluorescence emission over time of sample **3**. Emission at 524 nm after excitation at 465 nm of cpTMV-S23C-S65-*p*AF disk assemblies quantitatively labeled with Oregon Green 488 (**3**) is shown in blue and the instrument response function in red. The average fluorescence lifetime is well-described by a biexponential fit as shown by the fit residuals.

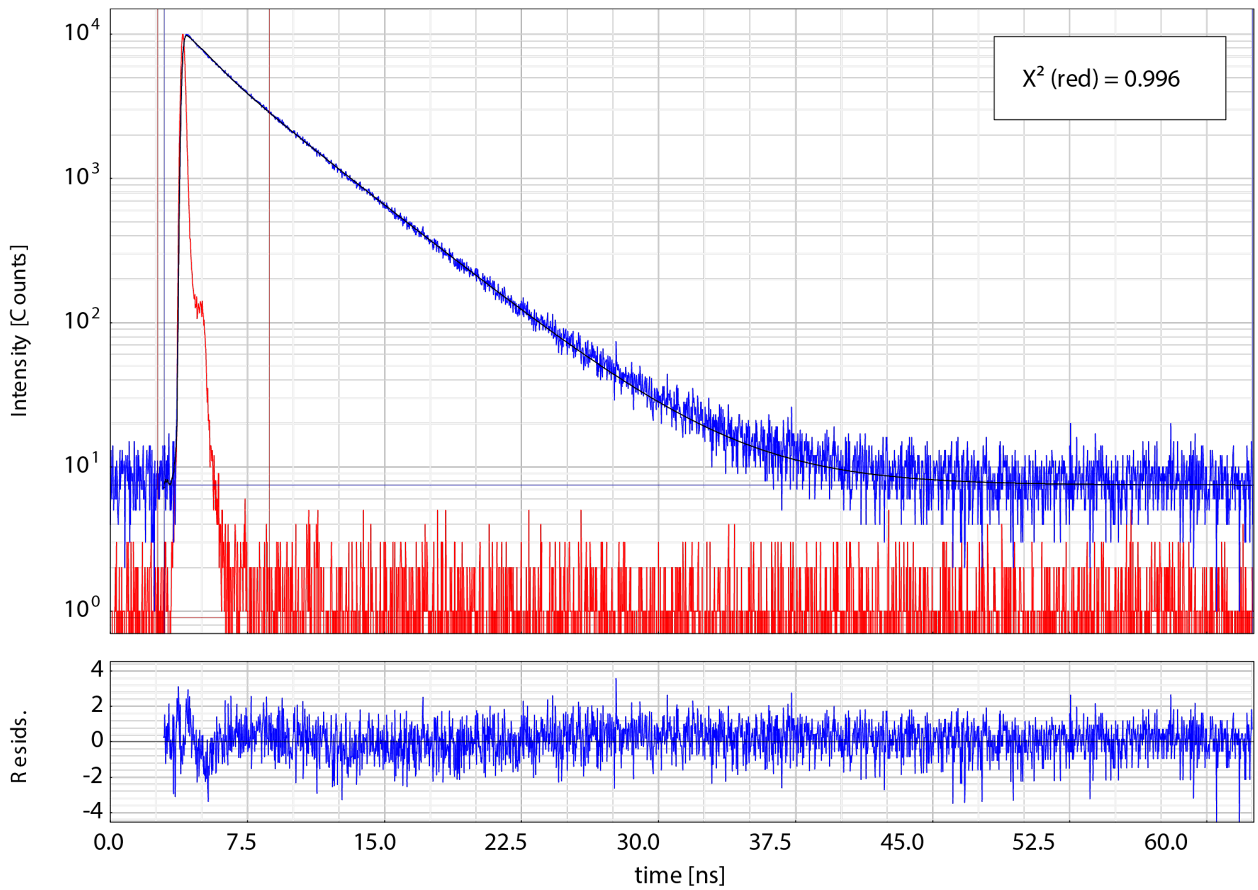

Figure S13. Fluorescence emission over time of sample **4**. Emission at 524 nm after excitation at 465 nm of cpTMV-S23C-S65-*p*AF disk assemblies quantitatively labeled with Oregon Green 488, as well as 4-methylcatechol at the S65-*p*AF position, of each monomer (**4**) is shown in blue and the instrument response function in red. The average fluorescence lifetime is well-described by a biexponential fit as shown by the fit residuals.

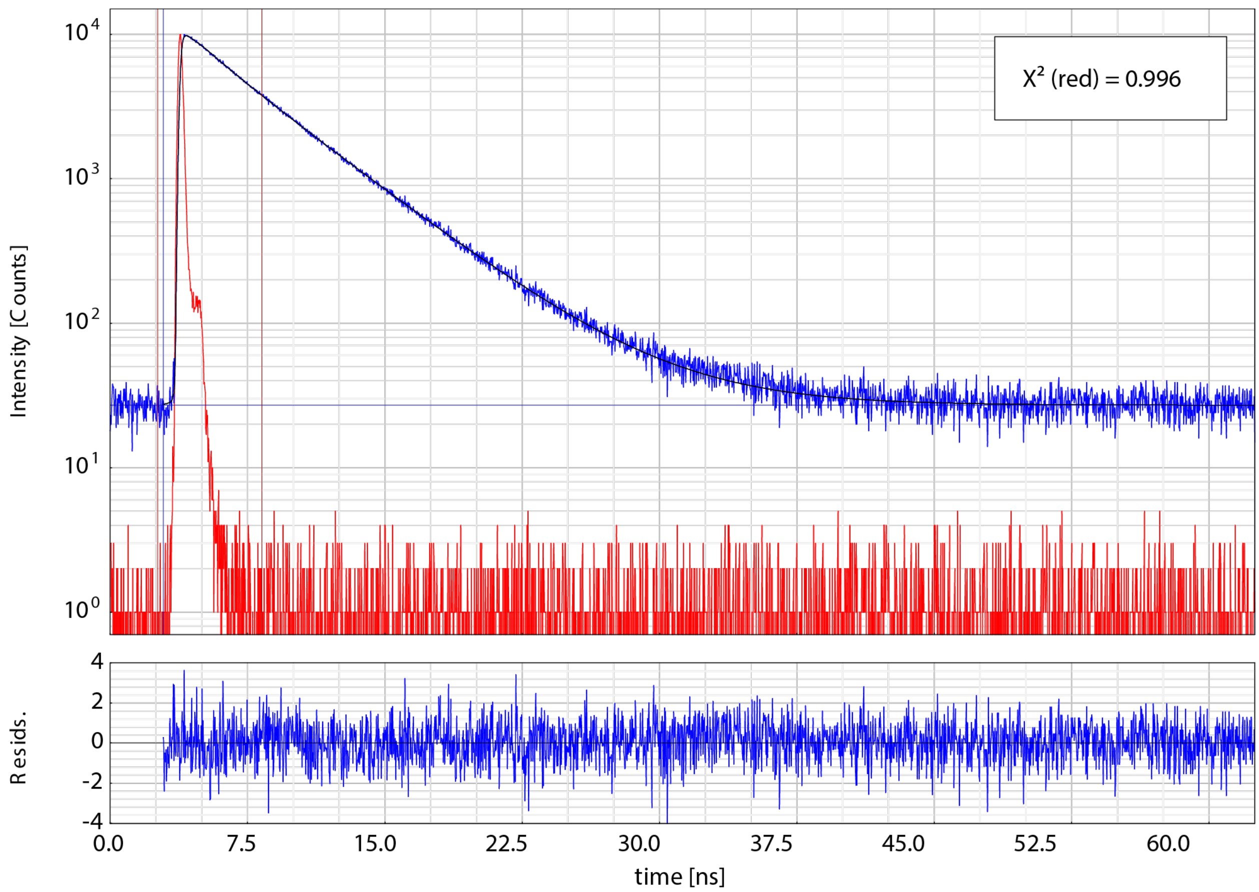

Figure S14. Fluorescence emission over time of sample **5**. Emission at 524 nm after excitation at 465 nm of cpTMV-S23C-S65-*p*AF disk assemblies quantitatively labeled with Oregon Green 488 and coupled to cpTMV-S65-3AY disks without any chromophore labeling (**5**) is shown in blue and the instrument response function in red. The average fluorescence lifetime is well-described by a biexponential fit as shown by the fit residuals.

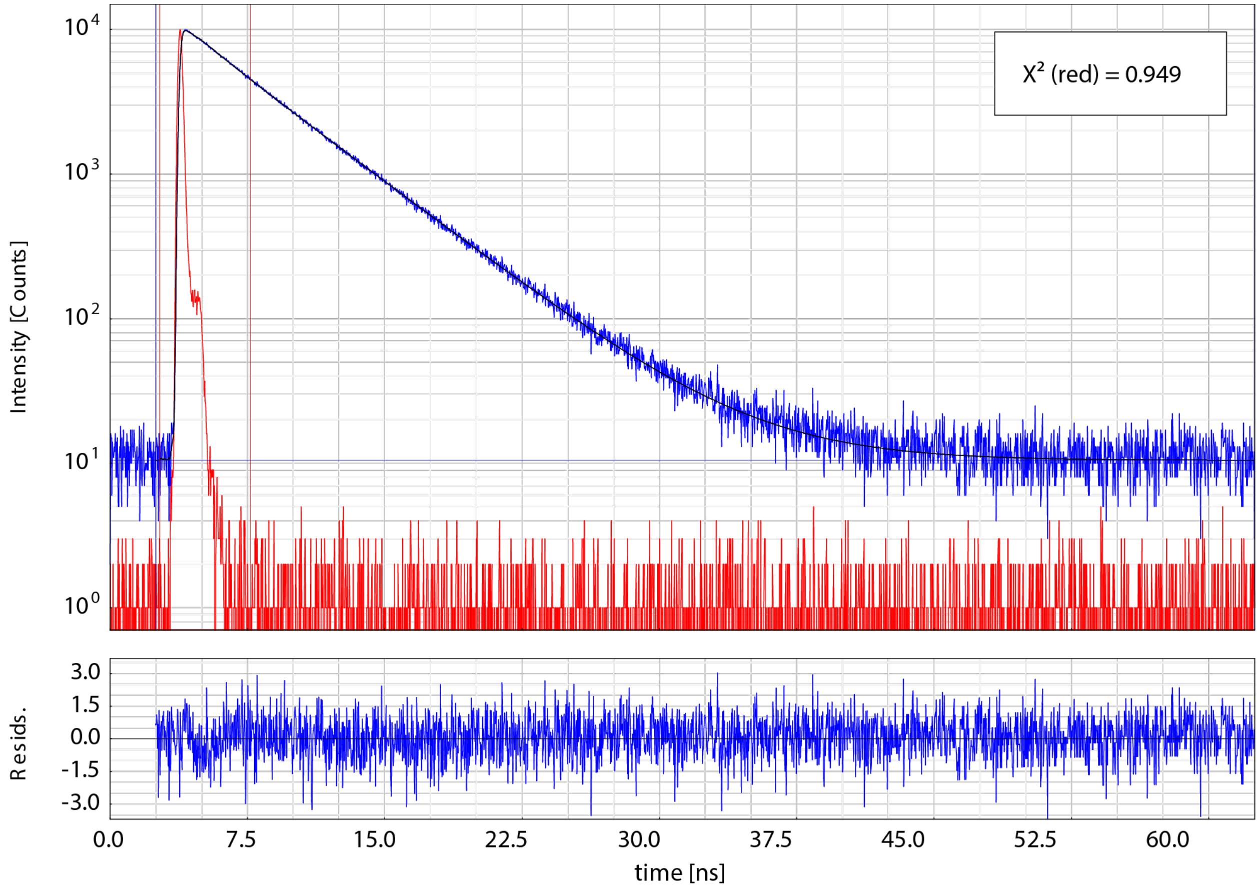

Figure S15. Fluorescence emission over time of sample **6**. Emission at 524 nm after excitation at 465 nm of cpTMV-S23C-S65-*p*AF disk assemblies quantitatively labeled with Oregon Green 488 and mixed, but not conjugated, with cpTMV-S23C-S65-3AY disks quantitatively labeled with Alexa Fluor 594 (**6**) is shown in blue and the instrument response function in red. The average fluorescence lifetime is well-described by a biexponential fit as shown by the fit residuals.

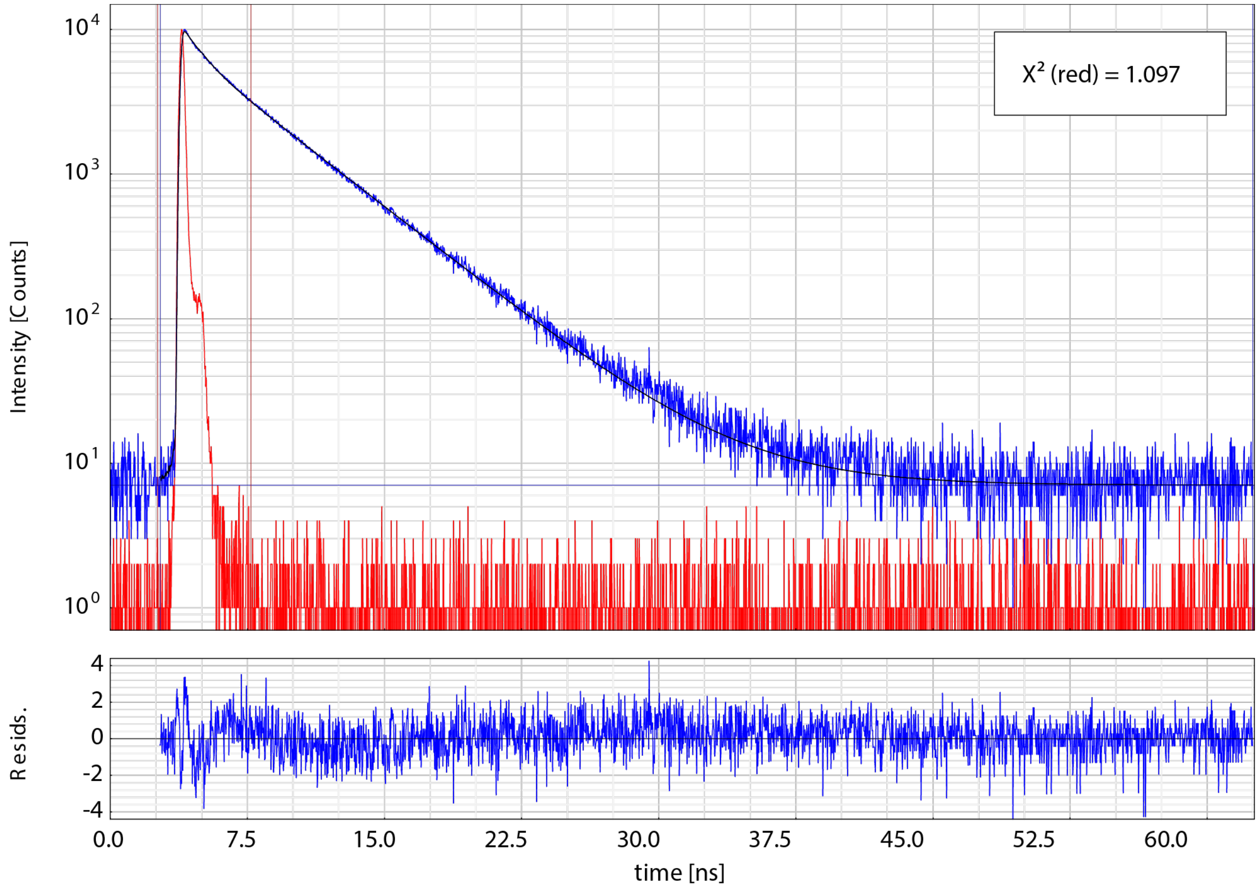

Figure S16. Fluorescence emission over time of sample **7**. Emission at 524 nm after excitation at 465 nm of cpTMV-S23C-S65-*p*AF disk assemblies quantitatively labeled with Oregon Green 488 and coupled to cpTMV-S23C-S65-3AY disks quantitatively labeled with Alexa Fluor 594 (**7**) is shown in blue and the instrument response function in red. The average fluorescence lifetime is well described by a biexponential fit as shown by the fit residuals.

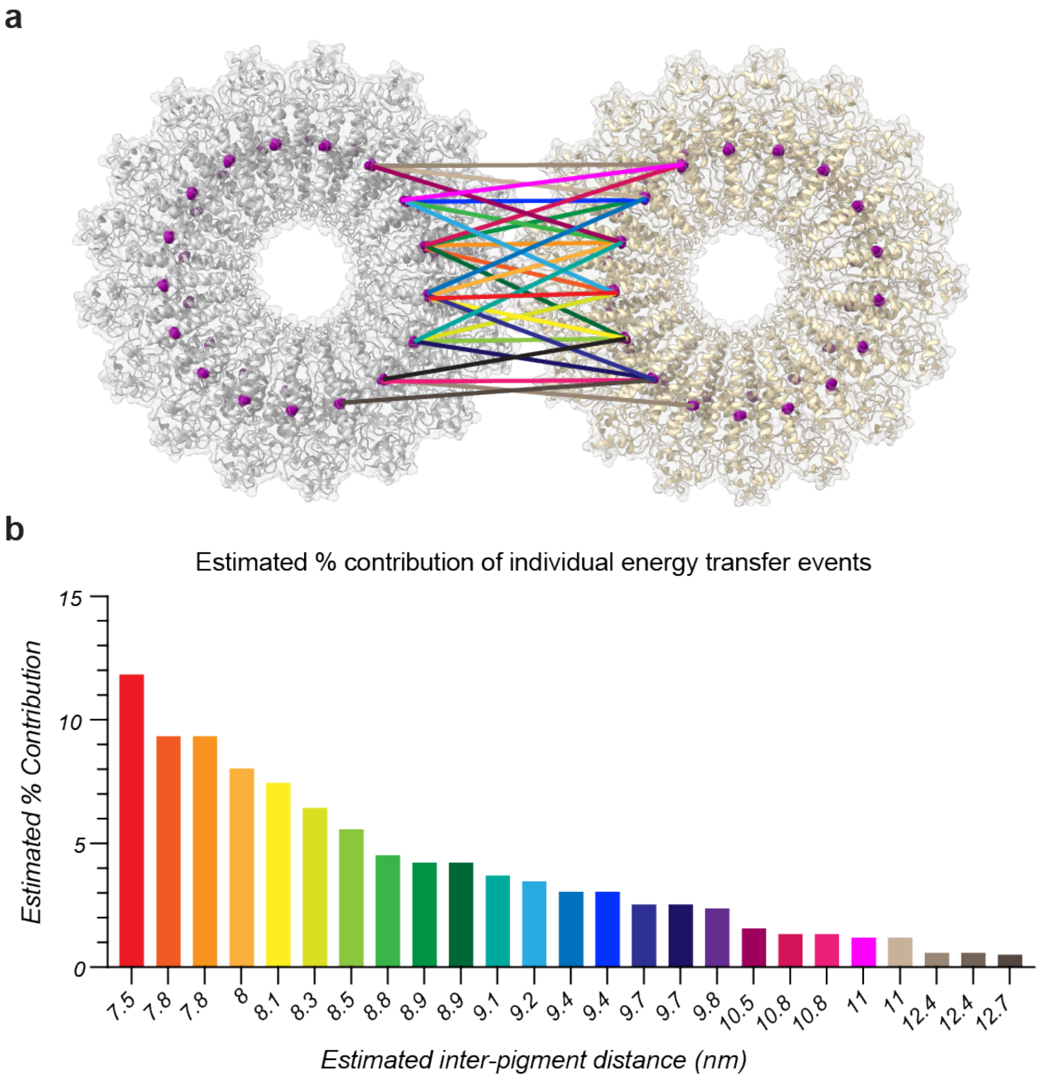

Figure S17. An estimate of the contributions of different donor–acceptor chromophore pairs in sample **7** to inter-disk energy transfer. (a) The distance between the chromophore attachment site (S23C) in two cpTMV disks in parallel in close proximity is shown for the closest pair (in red) and 24 additional pairs at increasing distances. This distance was used as a proxy for the inter-chromophore distances. (b) The distances between the chromophore attachment site (S23C) of the pairs shown in (a) and their estimated % contribution to inter-disk energy transfer based on the $1/R^6$ relationship between distance R and energy transfer timescale τ_T are shown, indicating that many redundant pathways are likely to significantly contribute to inter-disk energy transfer in this model. This is only an estimate, as the distance calculations are imprecise and orientational flexibility is not accounted for.
